# Marine biodiversity from zero to a thousand meters at Clipperton Atoll (Île de La Passion), Tropical Eastern Pacific

**DOI:** 10.7717/peerj.7279

**Published:** 2019-07-16

**Authors:** Alan M. Friedlander, Jonatha Giddens, Enric Ballesteros, Shmulik Blum, Eric K. Brown, Jennifer E. Caselle, Bradley Henning, Christian Jost, Pelayo Salinas-de-León, Enric Sala

**Affiliations:** 1 Pristine Seas, National Geographic Society, Washington, DC, United States of America; 2 Fisheries Ecology Research Lab, University of Hawai‘i, Honolulu, Hawai‘i, United States of America; 3 Centre d’Estudis Avançats de Blanes-CSIC, Blanes, Girona, Spain; 4 DeepSee, UnderSea Hunter Group, San José, Costa Rica; 5 Kalaupapa National Historic Park, US National Park Service, Kalaupapa, HI, USA; 6 National Park of American Samoa, US National Park Service, Pago Pago, American Samoa; 7 Marine Science Institute, University of California, Santa Barbara, Santa Barbara, CA, United States of America; 8 Exploration Technology, National Geographic Society, Washington, DC, United States of America; 9 Université de la Polynésie Française, Papeete, Tahiti, Polynésie Française; 10 Charles Darwin Research Station, Charles Darwin Foundation, Puerto Ayora, Galápagos, Ecuador

**Keywords:** Marine reserve, Marine biodiversity, Tropical Eastern Pacific, Clipperton Atoll, Deep sea, Coral reefs, Depth gradients, Mesophotic reefs, Île de La Passion, Oxygen minimum zone

## Abstract

Clipperton Atoll (Île de La Passion) is the only atoll in the Tropical Eastern Pacific (TEP) ecoregion and, owing to its isolation, possesses several endemic species and is likely an important stepping stone between Oceania, the remainder of the TEP, including other oceanic islands and the west coast of Central America. We describe the biodiversity at this remote atoll from shallow water to depths greater than one thousand meters using a mixture of technologies (SCUBA, stereo baited remote underwater video stations, manned submersible, and deep-sea drop cameras). Seventy-four unique taxa of invertebrates were identified during our expedition. The majority (70%) of these taxa were confined to the top 400 m and consisted mostly of sessile organisms. Decapod crustaceans and black corals (Antipatharia) had the broadest depth ranges, 100–1,497 m and 58–967 m, respectively. Decapods were correlated with the deepest depths, while hard corals were correlated with the shallow depths. There were 96 different fish taxa from 41 families and 15 orders, of which 70% were restricted to depths <200 m. While there was a decreasing trend in richness for both fish and invertebrate taxa with depth, these declines were not linear across the depth gradient. Instead, peaks in richness at ∼200 m and ∼750 m coincided with high turnover due to the appearance of new taxa and disappearance of other taxa within the community and is likely associated with the strong oxygen minimum zone that occurs within the region. The overall depth effect was stronger for fishes compared with invertebrates, which may reflect ecological preferences or differences in taxonomic resolution among groups. The creation of a no-take marine reserve 12 nautical miles around the atoll in 2016 will help conserve this unique and relatively intact ecosystem, which possesses high predator abundance.

## Introduction

**Figure 1:**
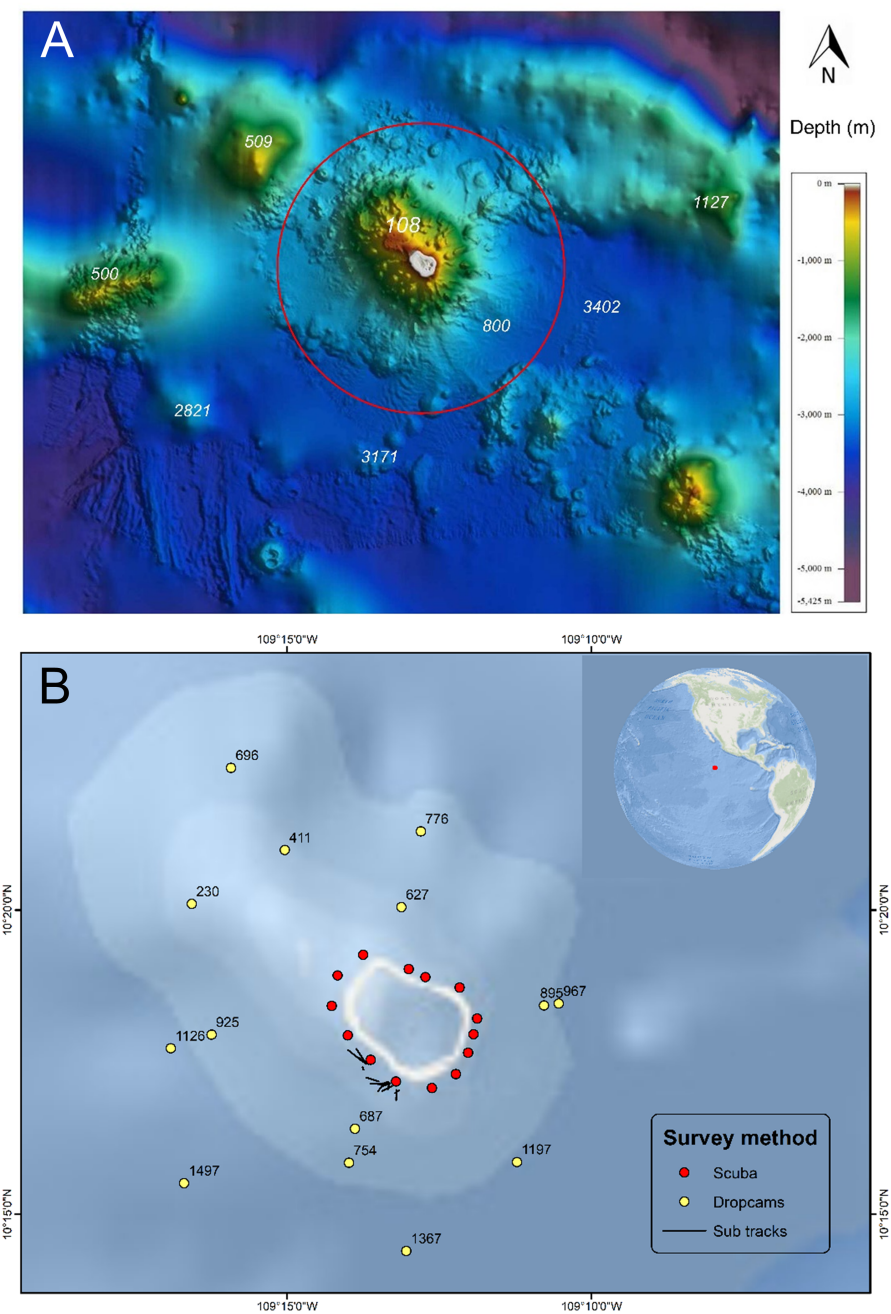
Bathymetry and sampling locations around Clipperton Atoll. (A) Bathymetry surrounding Clipperton Atoll.****Data from French Naval Hydrographic and Oceanographic Service (SHOM: Service hydrographique et océanographique de la Marine). Red circle is 12 nautical miles no-take reserve around the atoll. (B) Locations of underwater surveys. Numbers next to dropcam points are depths (m).

Clipperton Atoll (Île de la Passion) is an uninhabited French island in the Tropical Eastern Pacific (TEP) ecoregion (Spalding et al., 2007), located 1,080 km south-west of Mexico (Jost & Andréfouët, 2006; **Fig. 1**). It is the only atoll in the TEP, and with 3.7 km^2^ of coral-reef habitat, is the largest single coral reef in the region (Glynn, Veron & Wellington, 1996). The atoll has a highly eutrophic brackish-water lagoon with no outlet and a novel microbial community (Galand et al., 2012). Clipperton is an important location for seabirds, with the world’s largest breeding colony of masked boobies (*Sula dactylatra*) and the second largest brown booby (*S. leucogaster*) colony (Pitman, Ballance & Bost, 2005).

The atoll has been occupied at various times by guano miners, would-be settlers, or military personnel, mostly from Mexico, which claimed it until international arbitration awarded it to France in 1931 (Jost & Andréfouët, 2006). It was later occupied by the US military during WWII and is now under direct authority of the Ministry of Overseas France (Ministère des Outre-mer). Despite its remoteness, Clipperton is frequented by sport fishermen, as well as tuna and shark fishermen from various nations (Jost et al., 2015; Kroodsma et al., 2018).

The TEP is separated from the central and western Pacific by the East Pacific Rise, a divergent tectonic plate boundary on the Pacific floor running north to south that coincides with the boundaries of a unique biogeographic province with a high proportion of endemic species (Ekman, 1953; Allen, 2008). Owing to its isolation and western location in the TEP, Clipperton is likely an important stepping stone for dispersal of marine organisms between Oceania, the TEP, including other oceanic islands and the west coast of Central America (Emerson, 1994; Glynn, Veron & Wellington, 1996; Robertson & Allen, 1996; Lessios & Robertson, 2006). Within the TEP, Clipperton lies within the Ocean Island Province, which consists of five oceanic islands/archipelagos and possesses more transpacific species and more highly localized endemics compared to the mainland Cortez and Panamic provinces (Robertson & Cramer, 2009).

Several studies have been conducted on the shallow-water marine biodiversity of Clipperton, which have revealed a relatively depauperate flora and fauna (Allen & Robertson, 1997; Jost et al., 2015; Jost et al., 2016). In 2004–2005 a French-led expedition to Clipperton documented species from a wide range of taxonomic groups including: algae (83 species), corals (23), gastropods (70), bivalves (22), decapods (95), echinoderms (28), and fishes (163) (Salvat, Adjeroud & Charpy, 2008). A more recent and comprehensive checklist of cartilaginous and bony fishes from Clipperton yielded 197 species from 62 families (Fourriére et al., 2014). Most of these shallow-water organisms have affinities with the Indo-Pacific and central Pacific regions. Few efforts, however, have been made to describe the mesophotic coral ecosystems (MCEs) and deep-water habitats of this unique atoll, or across the TEP in general.

MCEs (30–150 m) are considered extensions of shallow reef communities and may greatly increase the available habitats for reef organisms, yet they have received relatively little attention owing to the difficulty in studying these habitats (Lesser, Slattery & Leichter, 2009; Puglise et al., 2009; Hinderstein et al., 2010; Rocha et al., 2018). Little is known about the deep-water communities within the region except for a few studies on slopes and seamounts around Isla del Coco (Costa Rica) to depths of 450 m using manned submersibles (Cortés & Blum, 2008; Starr, Green & Sala, 2012; Starr et al., 2012), and recent explorations at the Galápagos using Remote Operated Vehicles and manned submersibles to depths of 4,000 m (Carey et al., 2016).

The objective of this research was to describe the biodiversity at this remote atoll from shallow water to depths of over one thousand meters using a mixture of technologies to obtain a better understanding of the entire ecosystem. In addition, this work is intended to establish a baseline of ecosystem biodiversity and raise awareness of this unique location.

## Materials & Methods

### Site description

Clipperton Atoll is small (8.9 km^2^ including the lagoon), with an emergent land surface of only 1.7 km^2^, and a circumference of 11.8 km (Jost, 2003). It is surrounded by an almost continuous coral-rich fringing reef (ca. 50% live coral cover) and possesses high fish biomass (>3 t ha) (Ricart et al., 2016). The 50-m isobath is, on average, <500 m from shore (Glynn, Veron & Wellington, 1996).

### Data collection

In March 2016, we used a mixture of visual systems including underwater visual censuses by SCUBA, stereo baited remote underwater video stations (BRUVS), manned submersibles, and deep-sea drop cameras to assess the biodiversity of Clipperton Atoll to depths greater than one thousand meters. We did not survey cryptic biodiversity and limited collecting to a few samples taken with the manipulator arm of the submersible. All sampling was conducted during daylight hours (∼7:00 to 17:00). Permission to conduct this research was granted by the Haut-Commissariat de la République en Polynésie française (NO- HC/167/CAB/BSIRI/MG).

### Underwater visual census

Quantitative fish and benthic surveys were conducted at 14 sites around the atoll. Each site was surveyed at two depth strata (∼10 and 20 m), amounting to 28 site ×depth combinations. Three 25-m long transects were laid along isobaths within a homogeneous habitat, with 5 m separating each fish transect. At each survey site, a scuba diver counted and sized all fishes of total length (TL) ≥20 cm within a 4 m wide strip on an initial “swim out” while the transect line was laid (transect area = 100 m^2^), and all fishes of TL <20 cm were counted in a 2 m wide strip along the transect line on the way back (transect area = 50 m^2^). Total fish lengths were estimated to the nearest cm. All fishes were identified to the species level based on Robertson & Allen (2015).

Characterization of the benthos was carried out by divers on 50 m long transects laid parallel to the coast, with two depth strata (∼10 and 20 m). One 50-m transect was sampled at each depth strata. For algae, corals and other sessile macroinvertebrates, a point-intercept method was used along each transect. Species or taxa found every 20 cm on the tape measure were recorded. Echinoderms were quantified in 50 × 50 cm quadrats (25 random quadrats per depth stratum). Qualitative surveys of fishes and benthos were conducted at each survey site down to 40.8 m.

### Stereo Baited Remote Underwater Video Stations (s-BRUVS)

A stereo-video system consisting of two high definition cameras (GoPro Hero 4+) in waterproof housings (SeaGIS, Australia) was mounted on an aluminum A-frame and deployed in 20 m (Acuña Marrero et al., 2018). The frame was attached to a 20 kg weight with a buoy designed to keep the video system 1–1.5 m above the seabed. A bait bag filled with ∼1 kg of crushed tuna was attached to a 1.3 m long PVC pipe located in front of the cameras. A total of 24 BRUVS were deployed for a period of 90 min each around the perimeter of the atoll. BRUVS sampling was conducted over consecutive days from 14 to 20 March 2016, with an average number of deployments of 3.7 (±0.5 sd) per day. BRUVS were spaced at least 500 m apart to avoid overlap of individual camera bait plumes.

### Manned submersible dives

The *DeepSee* is a custom-built one-atmosphere submarine, capable of carrying a pilot and two passengers to a depth of 450 m (Cortés & Blum, 2008). The pilot and passengers are housed in a spherical acrylic compartment that provides an unrestricted 360-degree view. The submersible is equipped with a High-Definition digital video camera and a high-intensity-discharge lighting system. Invertebrates and fishes were identified to the lowest possible taxa and attributed to their observed depths using visual observations and video recordings during each submersible dive. A few samples of gorgonians were taken between 100 and 300 m with the manipulator arm of the submersible.

### Deep drop cameras

Deep Ocean Dropcams are high definition Sony Handycam HDR-XR520V 12-megapixel cameras encased in a borosilicate glass sphere and rated to a depth of 10,000 m (Turchik et al., 2015). Cameras were baited with ∼1 kg of frozen fish. The viewing area per frame was between 2–6 m^2^, depending on the slope of the substrate. Each individual organism documented within the video frame was identified to the lowest possible taxa.

### Statistical methods

Taxa were classified to the lowest possible taxonomic resolution across all taxonomic groups and methodologies. For invertebrates, this was mostly at the level of order or class, although some were only identifiable to phyla. Higher taxonomic levels included those organisms of the group that we were unable to assign to a lower level taxonomic rank. Shallow-water fishes were identified to species level, while deeper water species were only identifiable to family in many cases (McCosker & Rosenblatt, 2010; Robertson & Allen, 2015). All fishes were reclassified to family for analyses. All data were converted to presence at each depth observed. All observations were grouped into 20-m depth bins from 10 to 320 m, followed by 20-m bins at 420, 680, 740, 760, 880, 920, 960, 1120, 1180, 1360, and 1480 m.

We used Generalized Linear Mixed Effects Models (GLMMs) with a logit link to investigate fish family and invertebrate taxa probability of occurrence according to depth. Taxa were first grouped into 20 m depth bins, and then modeled as a binomial distribution with the statistical package lme4 (Bates et al., 2014). Models included a random effect to allow the relationship of the response variable to vary by depth. Therefore, the magnitude of the random effect quantified how community composition varied with depth. The significance of depth as a predictor of occurrence, and the significance of the relationship varying by taxon was assessed using a Likelihood Ratio Test (LRT) with the statistical package LmerTest (Bates et al., 2014). All data analyses were performed in the R programing environment, Version 1.0.136 (R Core Team, 2016).

Principal Coordinate Analysis (PCO) was used to investigate patterns in assemblage composition by depth with biplot vectors defining correlations overlaid on the ordinations. Bray Curtis similarity matrices of presence-absence data of invertebrates and fishes grouped into 20 m depth bins were used to conduct the PCO. Vectors defined correlations between invertebrates or fishes and the ordination and were calculated based on Spearman’s rank order correlation (*ρ*).

To investigate the change in community composition with depth, richness (number of taxa) and turnover (change in taxa) were calculated using the statistical package “codyn” in the R programing environment (Hallett et al., 2016). Taxon frequency of occurrence was grouped in 20 m depth bins, and three metrics of species turnover were calculated for the spatial gradient from 0–1,500 m depth: total turnover, appearances, and disappearances. Total turnover calculates the proportion of species that differ between depth points, while appearance and disappearance differentiate depths in which species appear vs. disappear, respectively (Hallett et al., 2016).

## Results

### Sample allocation

There were 14 sites sampled by four divers on open-circuit scuba to a maximum depth of 40.8 m (**Table 1**). BRUVS were performed at 24 locations around the atoll at a depth of 20 m for a total of 36 recording hours. We conducted 16 submersible dives ranging in duration from 2:00 to 2:48 hrs (total = 33.2 hrs). Maximum depths of each submersible dive ranged from 150 m to 330 m, with an average of 251 m (sd ± 59). Dropcams were deployed at 14 locations for 4 to 6.5 hrs (165 to 240 min of record-time), with an average record-time of 3 hrs. Deployment depths ranged from 230 m to 1,497 m ($\bar {X}=868.2 \pm  \mathrm{sd}~349.8$). Eight of the fourteen dropcam deployments occurred on soft bottom communities.

**Table 1:** Depth and total hours (hrs) for each survey method.

Method	*N*	Average depth m (± sd)	Min. depth (m)	Max. depth (m)	Total hrs
Open-circuit scuba	14	19.2 (7.7)	12.2	40.8	70.0^a^
BRUVS^b^	24	20.0	20.0	20.0	36.0
Submersible	16	250.6 (58.7)	0.0^c^	330.0	31.1
Dropcams	14	868.2 (349.8)	230.0	1497.0	40.0

**Notes.**

a Four divers per station—17.5 hrs total × 4 divers.

b BRUVS—Baited remote underwater video stations.

c Taxa were counted at the beginning of the dive as the sub descended.

### Invertebrates

**Figure 2:**
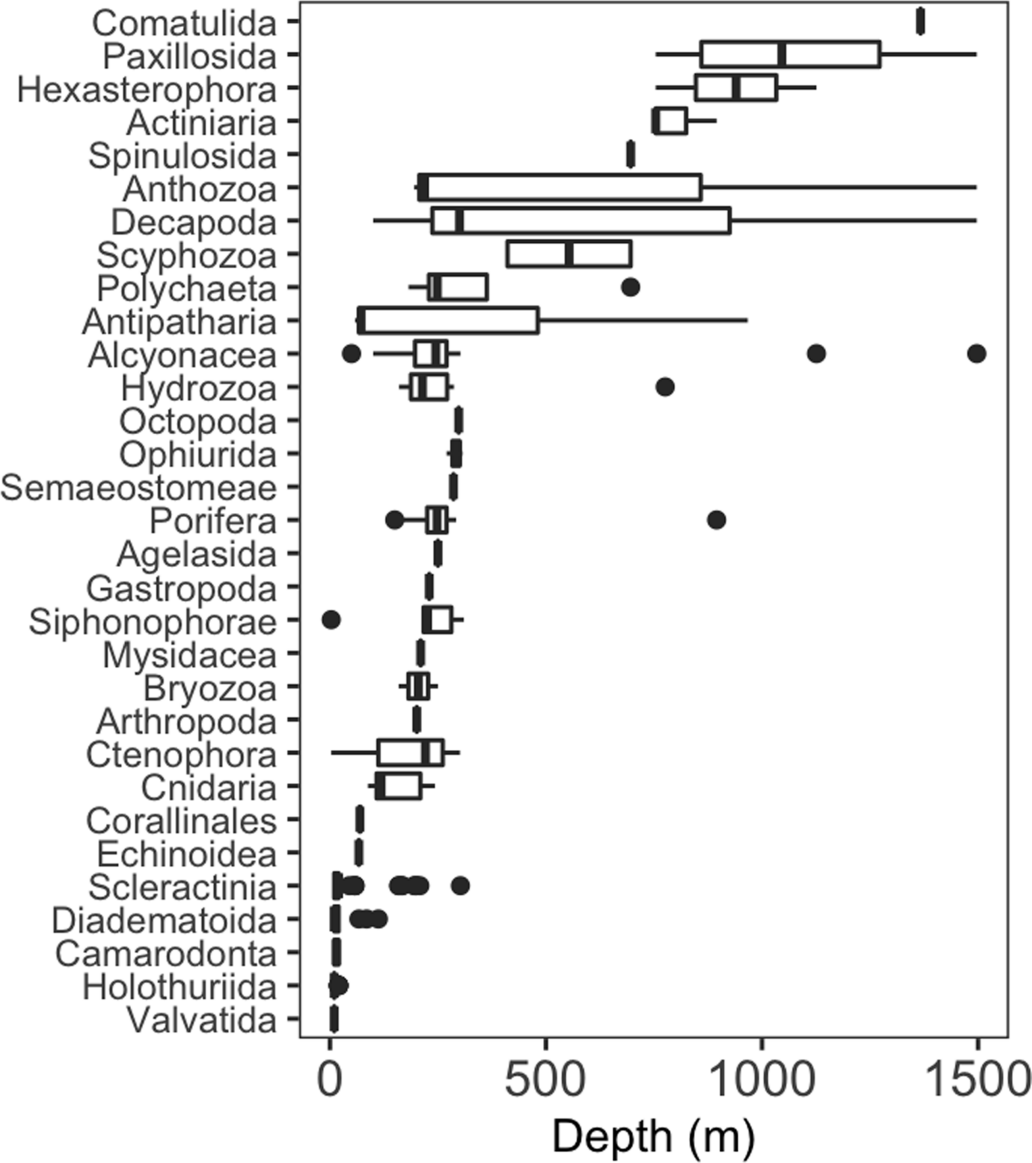
Depth distribution of invertebrate taxa by lowest possible taxon. Box plots showing median (vertical line), upper and lower quartiles, and 5th and 95th percentiles. Outliers are shown as dots.

Seventy-four different taxa of invertebrates were identified during surveys around Clipperton (Table S1). Taxa were grouped into order or the lowest possible taxonomic resolution for analyses, resulting in thirty high-level taxonomic categories. More than half of these higher-level taxa (52%) had a maximum depth of 310 m or less (**Fig. 2**). Most of these shallow groups had narrow depth ranges ($\bar {X}=148.7$ m ± 242.2 sd), excluding Hydrozoa, Porifera, and Alcyonacea, which had maximum depths of 776, 895, and 1,497 m, respectively. Decapoda and Antipatharia had the largest depth ranges, 100–1497 m and 58–967 m, respectively. Only two taxonomic groups (Hexactinellida and Comatulida) were restricted to depths >1,000 m.

**Figure 3:**
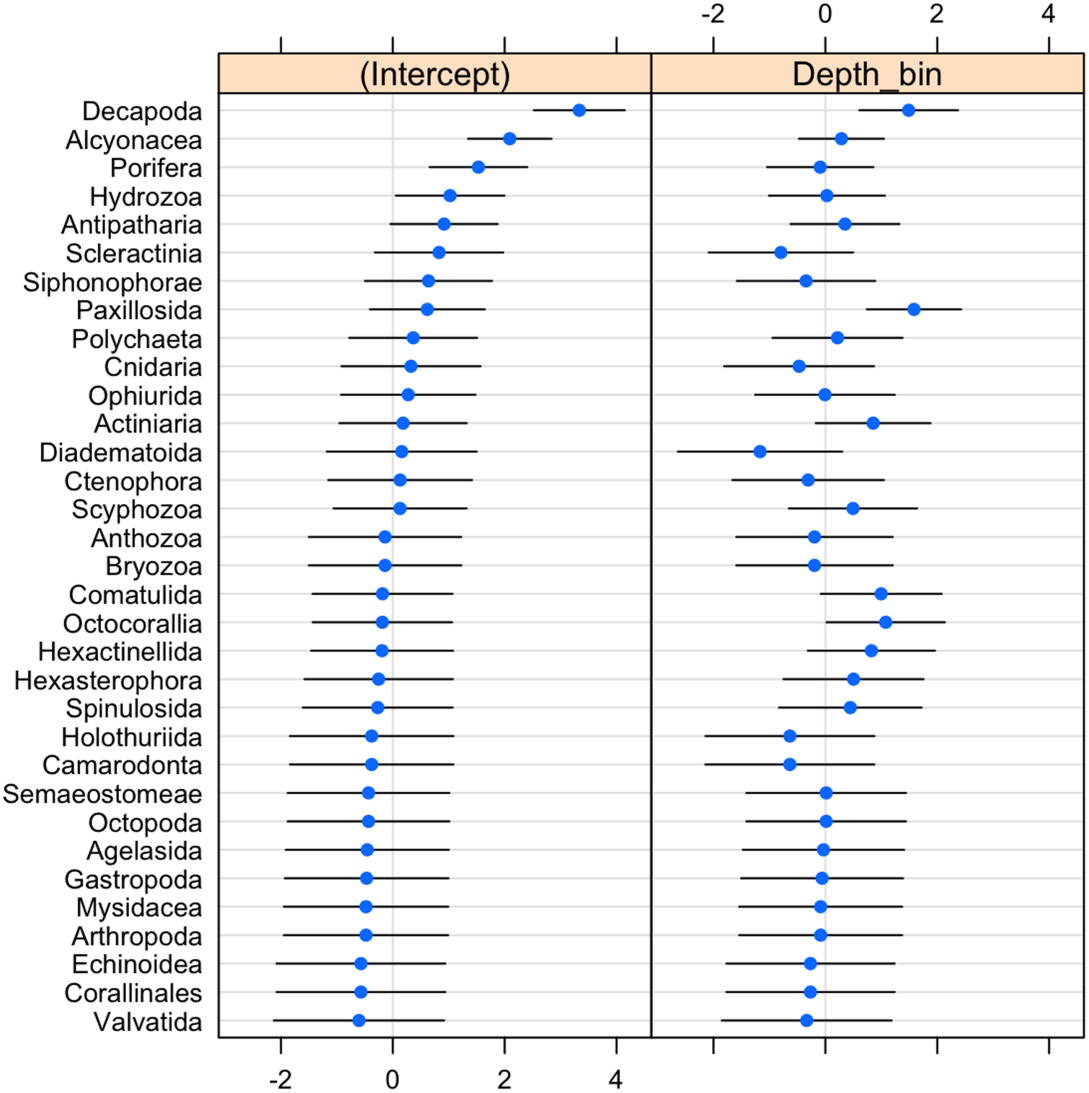
Dot-plot for GLMM of invertebrate taxonomic groupings by 20 m depth bins showing taxon-specific response to depth (model slopes on the *x*-axis), as well as the variation of response among groups. Groups are arranged by decreasing intercept.

The probability of invertebrate taxa occurrence declined with increasing depth (Fig. S1, *β* =  − 0.68, *se* = 0.27, *p* = 0.01). The significant negative relationship (LRT: *χ*^2^(1) = 15.64, *p* < 0.001) explained 43% of the variability in these data. Community composition varied significantly with depth (LRT: *χ*^2^(2) = 11.5, *p* = 0.003). Decapods such as crabs, shrimps, and lobsters along with sea stars (Paxillosida) responded positively to increasing depth, while stony corals (Scleractinia) responded negatively to depth (though the variability in slope overlapped with zero) (**Fig. 3**).

**Figure 4:**
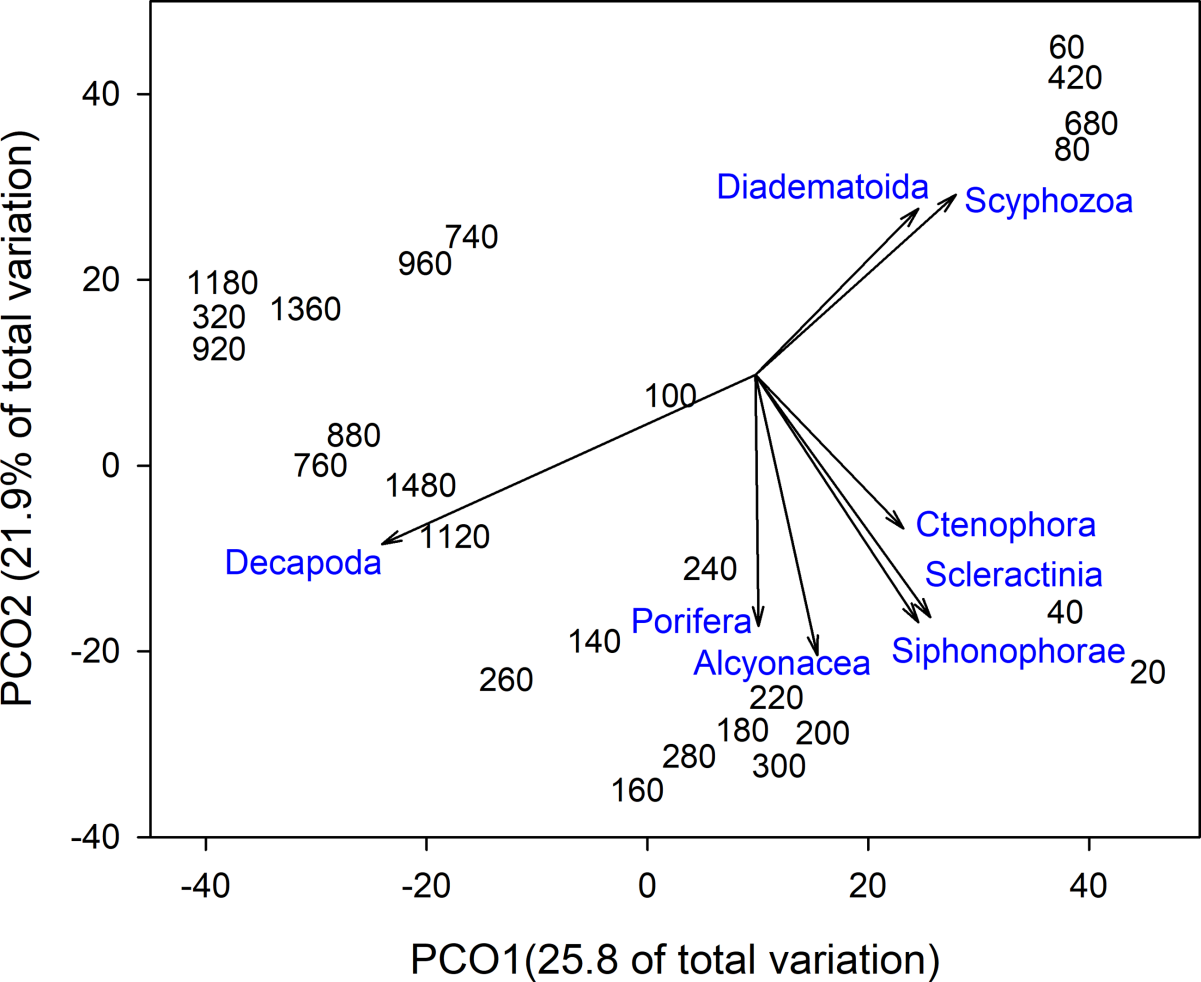
Principal Coordinates Analysis of invertebrate taxa presence-absence data using Bray Curtis similarity matrices of invertebrate taxa grouped into 20 m depth bins. Vectors defined correlations based on Spearman’s rank order correlation between invertebrate taxa and the ordination.

Invertebrate taxa separated out by depth along both axes in ordination space (**Fig. 4**). PCO1 explained 25.8% of the variation in assemblages, with Decapoda (*ρ* =  − 0.807) correlated with the deepest depths. Scyphozoa and Diadematoida were correlated with a mix of depth ranges (*ρ* = 0.427 and 0.350, respectively), while hard corals (Scleractinia, *ρ* = 0.366) and siphonophores (Siphonophorae, *ρ* = 0.321) were correlated with the shallow depths. PCO2 explained 21.9% of the variation in invertebrate taxa structure, with soft corals (Alcyonacea, *ρ* =  − 0.733), and sponges (Porifera, *ρ* =  − 0.656) correlated with the intermediate depth ranges from 140 to 300 m.

**Figure 5:**
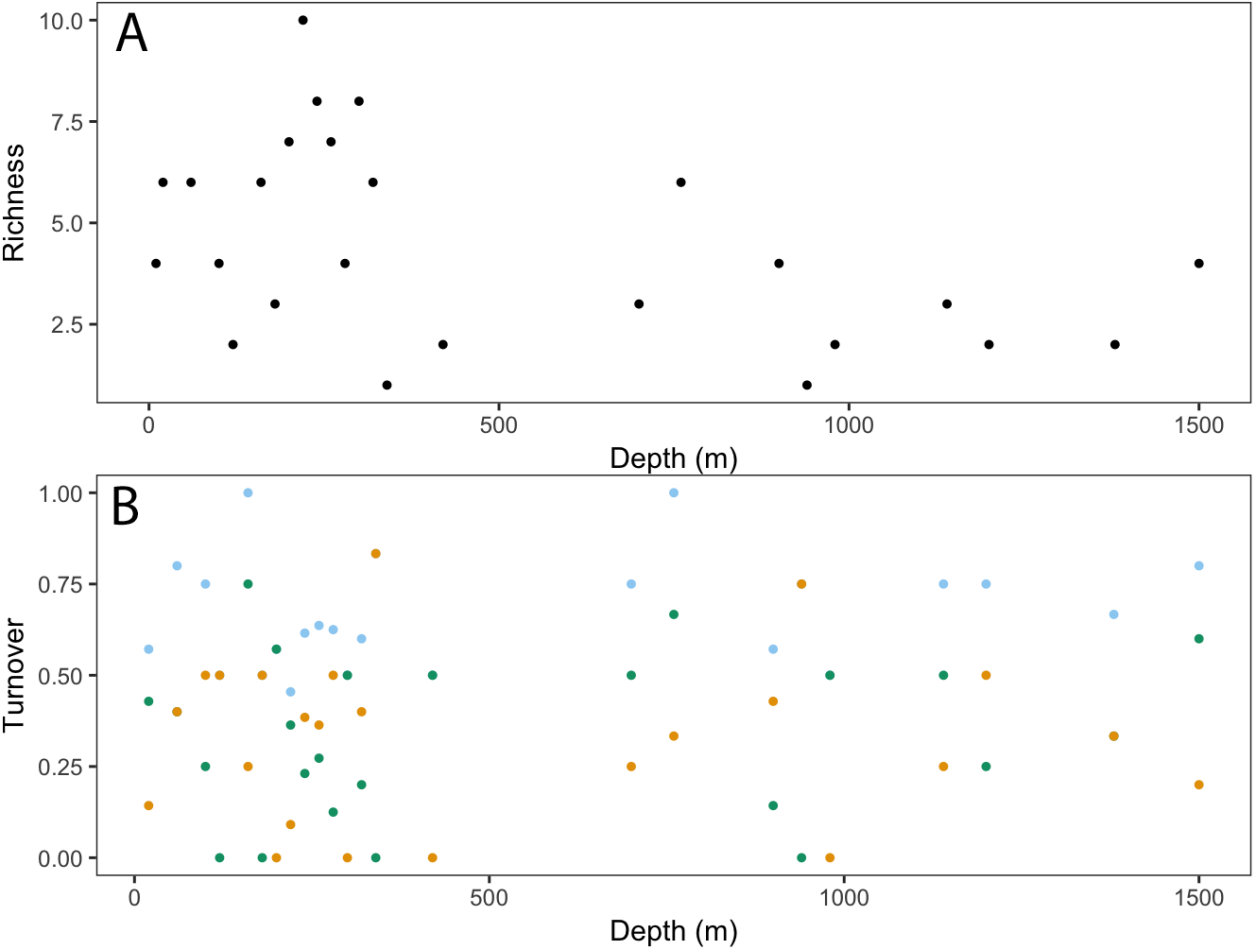
Invertebrate community patterns across depth. (A) Taxa richness; (B) turnover (total = light blue, appearances = dark green, disappearances = yellow) from 0–1,500 m depth at Clipperton Atoll.

Taxonomic richness (number of taxa) for invertebrates peaked at ∼200 m, following a relatively high turnover at ∼100 m (**Fig. 5**). This turnover was due to the appearance of new taxa observed with increasing depth. Richness continued to decline with depth, with a smaller second peak at ∼750 m. Again, this increase in richness coincided with a high turnover rate due to appearances of new taxa and the disappearance of others.

### Fishes

**Figure 6:**
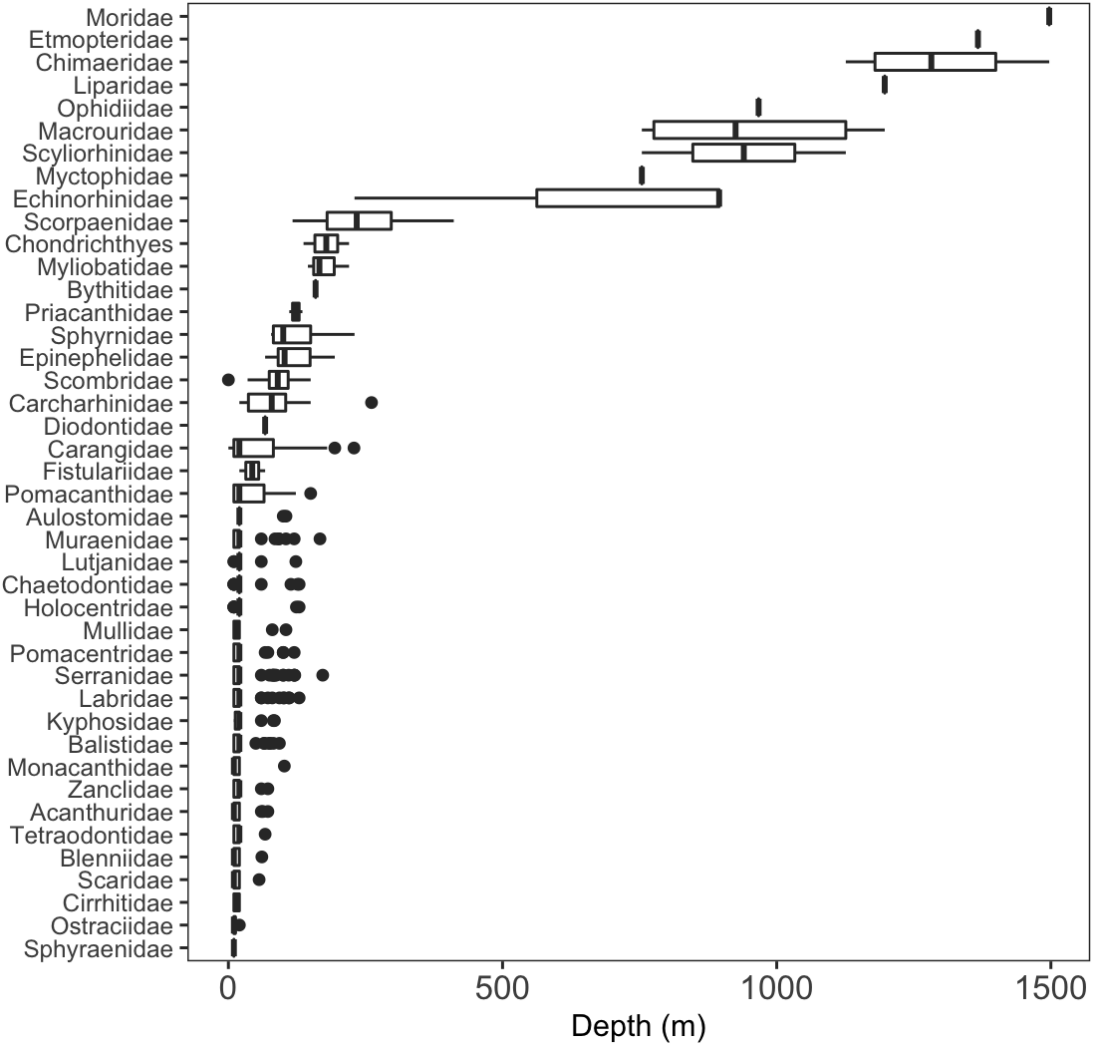
Depth distributions of fish families. A few sharks were only identifiable to class = Chondrichthyes.

**Figure 7:**
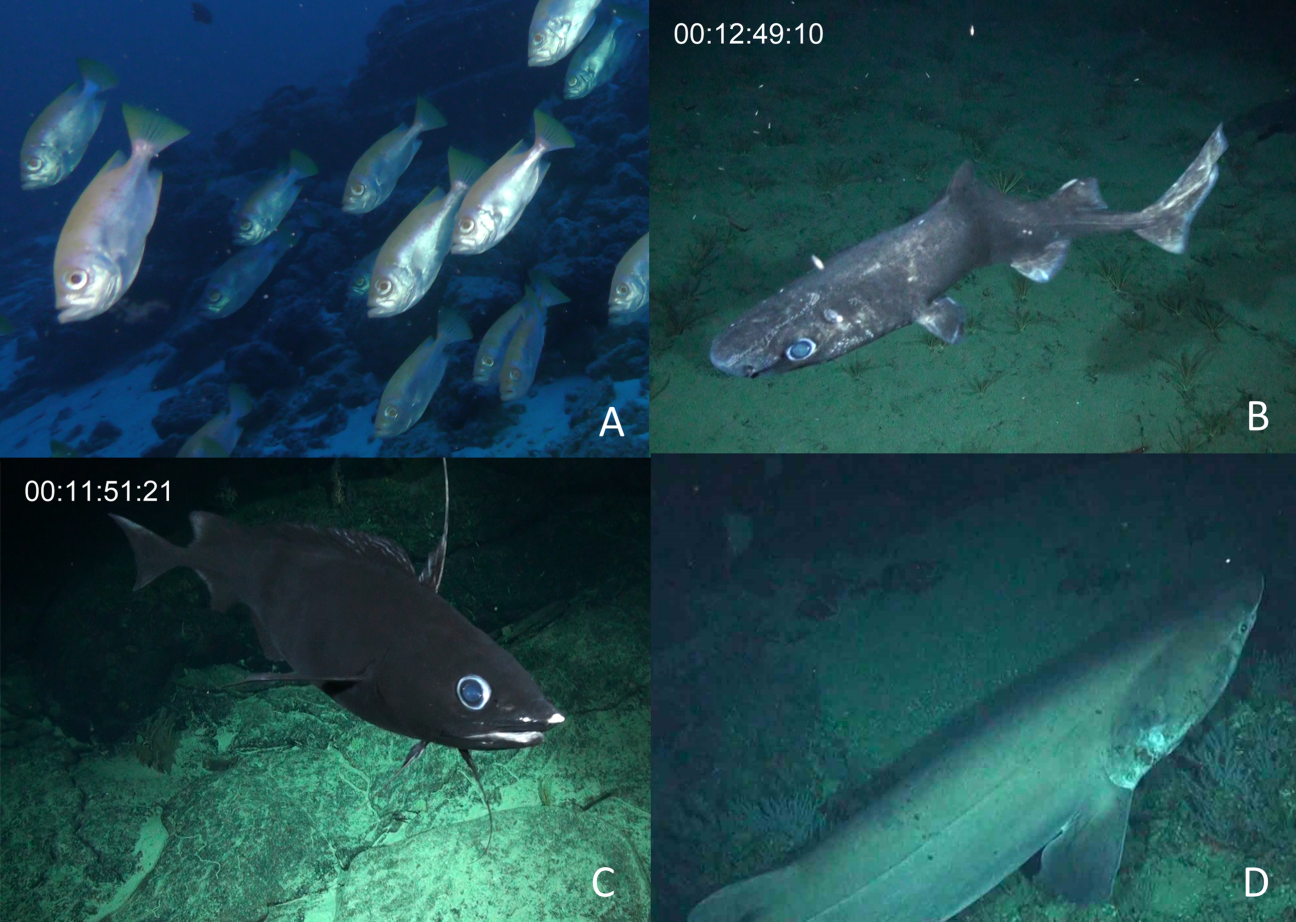
New fish taxa recorded at Clipperton. (A) *Cookeolus japonicus* (135 m), (B) *Etmopterus benchley i* (1,367), C. *Antimora* sp*.* (1,497 m), and D. *Echinorhinus cookei* (895 m).

We identified a total of 96 unique fish taxa from 41 fish families and 15 orders (**Fig. 6, Table S2). The most specious families were Carangidae (*n* = 7), Labridae (*n* = 7), and Serranidae (*n* = 7). We report 15 new records for fishes at Clipperton, including eight new families (Scyliorhinidae, Etmopteridae, Echinorhinidae, Chimaeridae, Macrouridae, Moridae, Bythitidae, and Liparidae). Most of these new records are from deep-water species (**Fig. 7, Fig. S2). Of the seven endemic fish species known from Clipperton, four were observed during our surveys. Of these, 67% were restricted to depths <200 m. Average depth ranges for fish families found in <200 m depth was 72.5 m (± 50.4 sd), and only slightly higher for those families in <400 m ($\bar {X}=84.4$ m ± 61.3 sd). Three families (Etmopteridae, Chimaeridae, and Moridae) were restricted to depths >1,000 m. Bramble sharks (Echinorhinidae) had the broadest depth distribution (230–895 m).

The probability of fish family occurrence declined with increasing depth (Fig. S3, *β* =  − 6.26, *se* = 1.12, *p* < 0.001). This negative relationship with depth was highly significant (LRT: *χ*^2^[1] = 34.76, *p* < 0.001), and explained 95% of the variability in the data (*R*^2^ = 0.95). Community composition also varied significantly with depth (LRT: *χ*^2^[2] = 147.02, *p* < 0.001). The magnitude of variation was 18.575 (*se* = 4.31) on a logit scale, indicating that the community composition changed entirely in terms of which fish families were present depending on depth.

**Figure 8:**
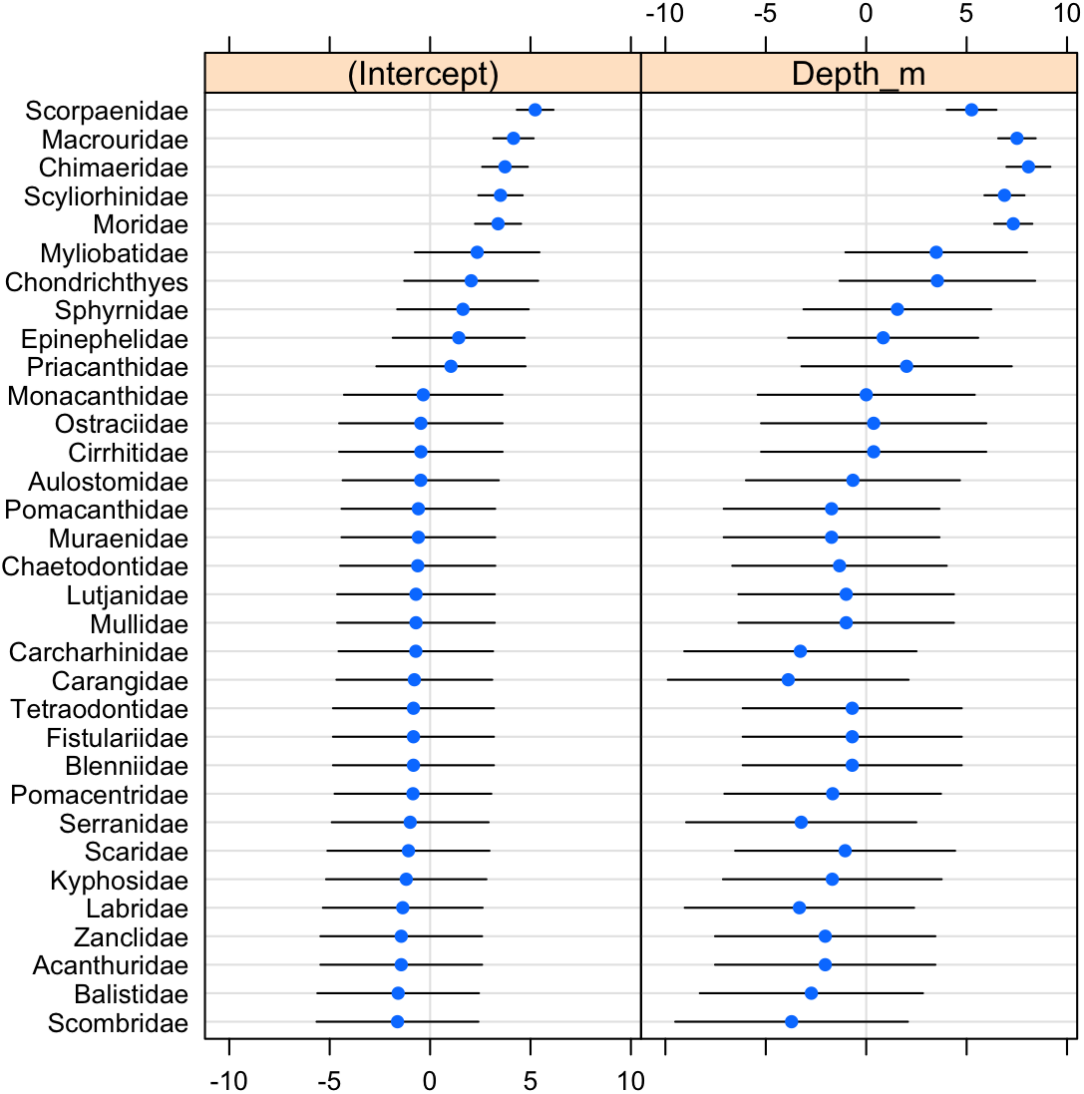
Dot-plot for GLMM of fish families by depth. The dot plot shows each fish family’s response to depth (model slopes on the x-axis), as well as the variation of response among families. Families are arranged by decreasing intercept.

The scorpionfishes (Scorpaenidae), grenadiers (Macrouridae), chimaeras (Chimaeridae), catsharks (Scyliorhinidae), and morids (Moridae) increased significantly in probability of occurrence with depth (**Fig. 8**). The majority (68%) of the fish families had a negative relationship to increasing depth, with requiem sharks (Carcharhinidae), jacks (Carangidae), groupers (Serranidae), wrasses (labridae), and tunas and mackerels (Scombridae) showing the strongest relationships. However, except for the five deep-water families noted above, the depth distributions of all other families were highly variable as evidenced by the modelled slopes overlapping with zero.

**Figure 9:**
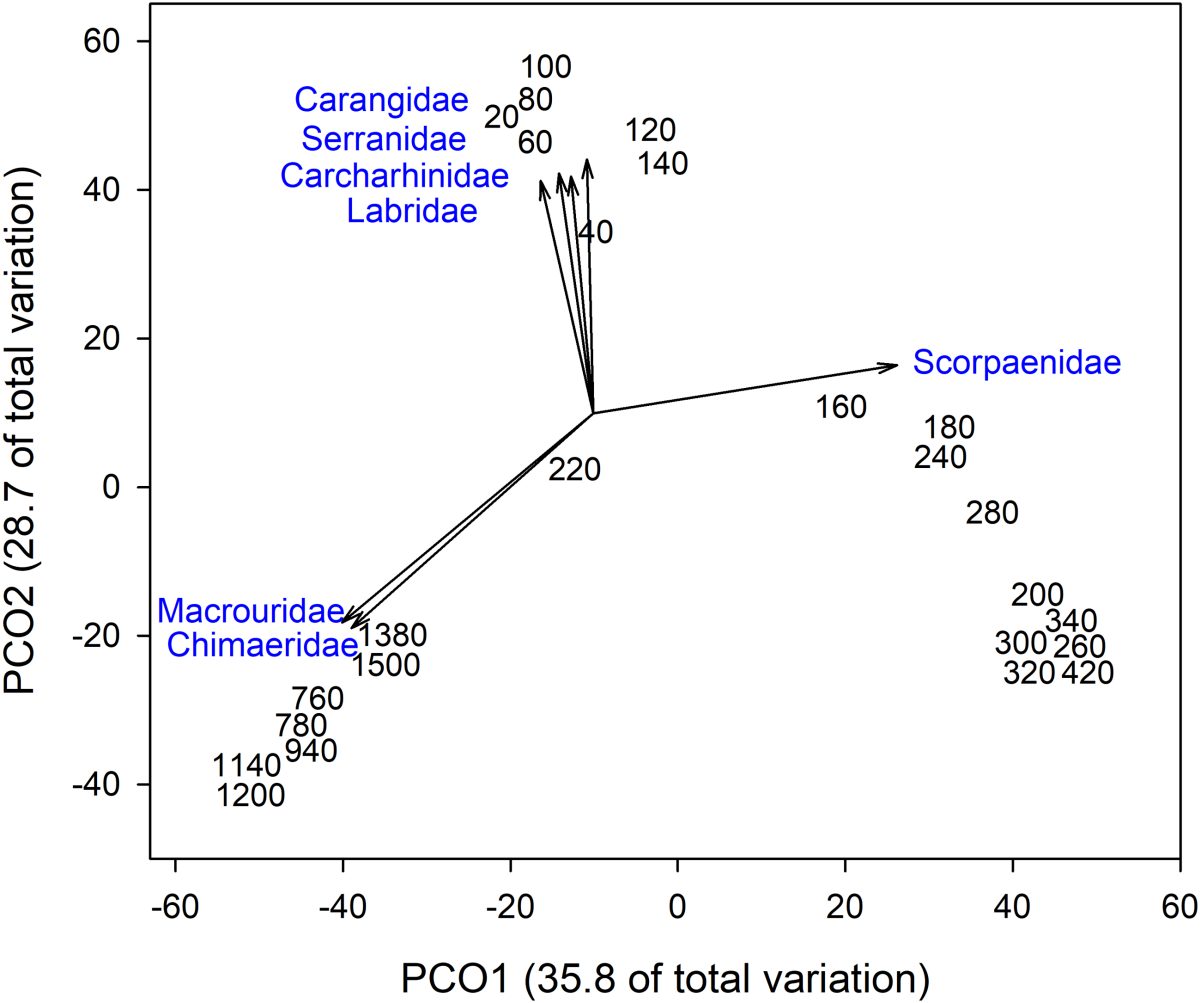
Principal Coordinates Analysis of fish family presence-absence data using Bray Curtis similarity matrices of fish families grouped into 20 m depth bins. Vectors defined correlations based on Spearman’s rank order correlation between fish families and the ordination.

**Figure 10:**
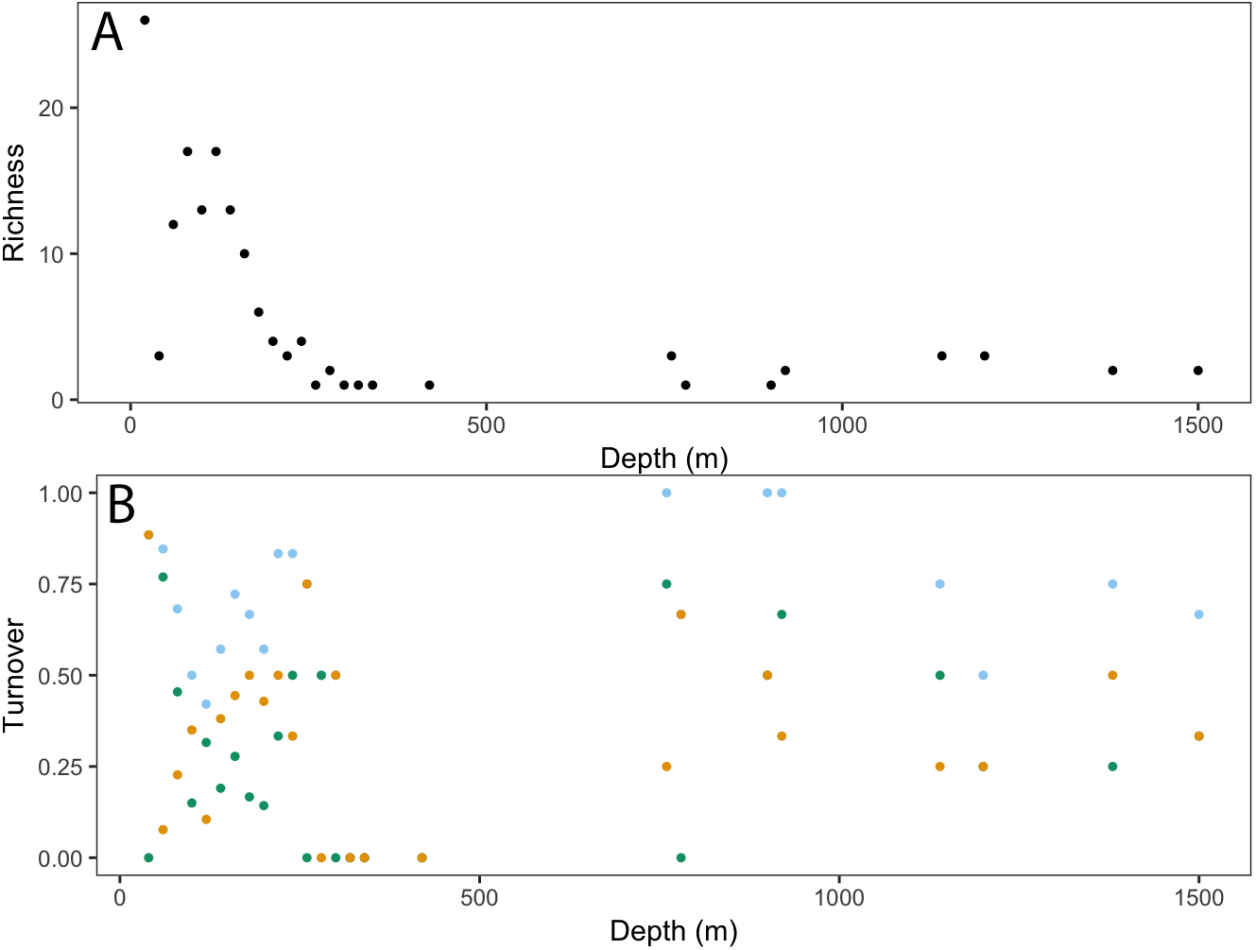
Fish community patterns across depth: A: Species richness, B: turnover (total = light blue, appearances = dark green, disappearances = yellow) from 0–1,500 m depth at Clipperton Atoll.

Analysis of fish assemblages showed clear separation in ordination space, with three major depth clusters (20–140, 160–420, 760–1,500, **Fig. 9**). PCO1 explained 35.8% of the variation in assemblages, with grenadiers (Macrouridae, *ρ* =  − 0.698) and chimeras (Chimaeridae, *ρ* =  − 0.548) correlated with the deepest depth cluster, and scorpionfishes (Scorpaenidae) correlated with the middle depth stratum (*ρ* = 0.872). PCO2 explained 28.7% of the variation in fish assemblage structure, with jacks (Carangidae, *ρ* = 0.815), groupers (Serranidae, *ρ* = 0.771), sharks (Carcharhinidae, *ρ* = 0.766), and wrasses (Labridae, *ρ* = 0.746) correlated with the shallow depth cluster. This shallow cluster was somewhat disconcordant, with the deeper depth bins separated from the shallower ones. Fish assemblage richness declined rapidly with depth, with slight increases at ∼100–200 m, ∼750 m, and at ∼1,200 m (**Fig. 10**).

## Discussion

This study represents the first examination of the biodiversity at a remote atoll from shallow-water to depths greater than one thousand meters using a mixture of survey methods and technologies. While there is a long history of research conducted at Clipperton (Snodgrass & Heller, 1904; Hertlein et al., 1957), most studies have focused on the shallow-water ecosystems (Glynn, Veron & Wellington, 1996; Jost et al., 2015; Fourriére et al., 2014; Ricart et al., 2016). As a result, the MCE and deep-water habitats of the atoll had not been well described prior to our expedition. Our integrated approach sheds new light on the distribution of marine communities across a large depth gradient at the only coral atoll in the TEP and represents one of the first studies to integrate multiple methods to characterize an ecosystem from nearshore depths to nearly 1,500 m.

While there is a decreasing trend in taxa richness for both fishes and invertebrates with increasing depth, these declines are not linear across the depth gradient. Instead, peaks in richness were observed at ∼200 m and 750 m, which also coincided with higher assemblage turnovers at these depths. Fish assemblages showed three major depth clusters (20–140 m, 160–420 m, 760–1,500 m), and these align somewhat with observed patterns of richness.

The overall depth effect was stronger for fish families than for invertebrate taxonomic groups, which may reflect physiological constrains (i.e., the influence of the oxygen-minimum zones - OMZ), substrate differences, ecological preferences, or the differences in taxonomic resolution owing to our limited ability to identify taxa from visual methods, particularly for deeper water taxa, where taxonomy is typically poorly resolved (Figs. S1 and S3). Although little is known about the biodiversity of deep-sea ecosystems of the TEP, at Isla del Coco National Park in Costa Rica, sharp faunal changes were reported at relatively shallow depths (∼50 m) during quantitative submersible dives to 450 m (Cortés & Blum, 2008). Similarly, underwater surveys of reef fish assemblages and coral communities in the Pacific and western Atlantic showed high dissimilarity between shallow (altiphotic) and mesophotic depth strata (Rocha et al., 2018). Olavo et al. (2011) proposed a faunal corridor for species associated with deep reef formations along the shelf-edge zone (40–200 m) of the South American continental margin, connecting the south-western Atlantic and the Caribbean provinces. In the Caribbean, submersible-based surveys revealed a unique reef-fish assemblage below the mesophotic zone between ∼130 and 309 m (rariphotic zone) that was taxonomically distinct from shallower faunas (Baldwin, Tornabene & Robertson, 2018). We observed a similar break in community structure between the mesophotic and rariphotic zones at Clipperton. Unlike Baldwin, Tornabene & Robertson (2018), who found that the rariphotic assemblage was dominated by deep representatives of typical shallow taxa, the rariphotic assemblage at Clipperton was dominated almost exclusively by scorpaenids. Our sampling was conducted at a much broader scale than the studies noted above, which may have limited our ability to detect differences in community structure in the shallower depths (<140 m).

At Tristan da Cunha in the South Atlantic, Caselle et al. (2018) found distinct differences in the deep fish community above and below ∼750 m, which was correlated with discontinuities in temperature and salinity at this depth. Similarly, characterization of the demersal fishes on the continental slopes of New Zealand using baited cameras also found that species richness peaked in shallow water, followed by a decrease beyond 100 m to a stable average from 700 to 1,200 m (Zintzen et al., 2012). The regional-scale distribution of benthic invertebrate megafauna along the Western Australian continental shelf down to >1,000 m is influenced by transitions in bottom temperature and oxygen concentration, which are associated with the warmer, oxygen-poor tropical waters of the southward flowing Leeuwin Current, the northward-flowing, colder, oxygen-rich Leeuwin undercurrent at 200–400 m, and Antarctic Intermediate Water below 400 m (Williams et al., 2010).

The geographic and vertical distribution of many species may be restricted by the presence of oxygen-minimum zones (OMZ), which are defined as oxygen concentrations <0.5 ml l^−1^ (Rogers, 2000). Several oceanic regions around the world have large, persistent oxygen minima, including the TEP (Karstensen, Stramma & Visbeck, 2008). The OMZ has its largest westward extension in the tropical North Pacific at around 10° N, with oxygen values of <50 µmol kg^−1^ covering almost the entire area below about 100 m depth (Karstensen, Stramma & Visbeck, 2008). The OMZ has an influence on the abundance and diversity of species, with the upper and lower boundaries of the OMZ being locations of increased biogeochemical activity. Elevated zooplankton abundances and a peak in benthic megafauna and macrofauna abundance was observed at the lower depth range of the OMZ (∼740–850 m) elsewhere in the TEP (Levin, 1991; Wishner et al., 1995). There is evidence for increasing dissolved oxygen depletion and vertical expansion of the OMZ worldwide because of global climate change (Stramma et al., 2008; Hofmann & Schellnhuber, 2009; Keeling, Körtzinger & Gruber, 2010), and this could greatly impact the deep-sea biodiversity around Clipperton in the future. This present study can serve as a baseline to monitor changes in benthic biodiversity across depth gradients at Clipperton and elsewhere in the TEP. Studies such as ours are needed because some species are predicted to move deeper with increasing ocean temperatures, along with many unpredictable changes to the ecosystem.

Clipperton’s shallow-water ecosystem is biogeographically unique in the TEP (Ekman, 1953; Allen, 2008). The remoteness and small area of the atoll has resulted in low genetic diversity and apparent kinship for two endemic fish species (Crane et al., 2018). Of the seven endemic fish species known from Clipperton, four were observed during our surveys. The endemic Clipperton angelfish (*Holacanthus limbaughi*) is considered a shallow-water species; however, recent submersible dives have found large aggregations (>100 individuals) at depths of 150 m (Clua et al., 2019), thus highlighting the connectivity between shallow and deeper water habitats. Previous studies on the shallow-water marine biodiversity of Clipperton have provided evidence to suggest that it may also be an important stepping-stone between Oceania, the TEP, and the coast of Central America (Glynn, Veron & Wellington, 1996; Emerson, 1994; Robertson & Allen, 1996). We report 15 new records for fishes at Clipperton, most of which are deep-water species, which have not previously been well surveyed at Clipperton. While the shallow water ecosystem at Clipperton is unique and is a potential link with other locations within the region, these deeper depths may be an important extension of this distinctive ecosystem as well.

By exploring deeper habitats, this study helps put Clipperton into the larger context of biodiversity value, especially as a link to other isolated coral reef ecosystems in the Eastern Pacific. The olive grouper, *Epinephelus cifuentesi*, was only described in 1993 and is a deep-water species (40–135 m) with a range restricted to the tip of Baja, southern Mexico to Ecuador, Galápagos Islands, Isla del Coco, Revillagigedo, and Alijos rocks (Craig, Sadovy de Mitcheson & Heemstra, 2011; Rocha et al., 2013). Olive grouper were once a major component of the deep-water grouper fishery in the Galápagos, but stocks have declined dramatically there due to overfishing and the species is currently rare elsewhere in its range, leading to its listing as Near Threatened by IUCN (Schiller et al., 2015). Large olive groupers were relatively abundant and occurred on 44% of our submersible dives at Clipperton. This is the first record for this species at Clipperton and its prevalence at the atoll may suggest an important refuge and potential source population for this overexploited and rare species.

Clipperton is also intriguing from the perspective of the maintenance of biodiversity on a small and remote island. From a conservation perspective, small and remote islands like Clipperton are predicted to suffer a greater loss of biodiversity if immigration is low or reduced, even if niche mechanisms are intact (Chisholm et al., 2016). Therefore, changes in oceanographic conditions can increase extinction risks for species with restricted ranges and/or low densities, as is typical for the ecosystems of small isolated islands such as Clipperton. The fire coral (*Millepora exaesa*) was previously reported from Clipperton (Glynn, Veron & Wellington, 1996; Charpy, 2009) but was absent from our surveys, highlighting the episodic nature of the ecosystem.

The Eastern Pacific Barrier greatly limits connectivity between the TEP and the central Pacific and the long-term persistence of populations depends on local recruitment within the region (Romero-Torres et al., 2018). Although bidirectional dispersal pathways exist between Clipperton and the Line Islands, and to a lesser extent Hawai‘i, this low frequency and weak connection suggest that populations are more vulnerable to disturbance than previously thought (Romero-Torres, Acosta & Treml, 2017; Romero-Torres et al., 2018). The maintenance of stepping stones like Clipperton is critical for the preservation of long-distance connectivity across the TEP and with the Central Pacific (Romero-Torres et al., 2018).

Deep-sea mineral companies are particularly interested in the exploration and exploitation of the rich mineral deposits of the Clarion-Clipperton Fracture Zone (CCZ), adjacent to Clipperton’s EEZ (Wedding et al., 2013; Wedding et al., 2015; Voosen, 2019). France has not yet exploited the deep minerals in Clipperton’s EEZ; however, a French-Mexican oceanographic survey in 1997 found polymetallic nodules within the EEZ (Jost et al., 2015). These deep-sea communities have low productivity, delicate biogenic habitat structure, and very low recovery rates from physical disturbance, and as such are particularly vulnerable to human influences (Smith & Demopoulos, 2003; Smith et al., 2008a; Smith et al., 2008b; Miljutin et al., 2011).

During our expedition, we observed abandoned longline gear on every dive, as well as line, nets, rafts, radio beacons and fish aggregation devices (FADs) scattered along the beach. FADs have commonly washed ashore at Clipperton, with at least 14 in 2015, and 5 in 2016 (Jost et al., 2016). Due to absence of a permanent French presence on the island and only intermittent patrols, authorized (48 Mexican ships in 2017) and non-authorized seiners and longliners are frequently seen fishing around Clipperton. The near absence of adult sharks observed during our expedition (Jost et al., 2016), which included 36 h of baited camera surveys (BRUVS), is likely the result of both targeted fishing and bycatch interactions. While early reports refer to the abundance of sharks at Clipperton (Limbaugh, 1963; Skaggs, 1989), Allen & Robertson (1997) reported that they were surprising uncommon in 1994 and noted that intensive fishing had removed ∼2,000 sharks from the area the previous year.

Another indicator of extensive fishing pressure around Clipperton is the drastic decline in the population of masked boobies in the past decade from 110,000 to 40,000 individuals (Jost et al., 2016). Seabirds depend on tuna to drive baitfish to the surface for them to feed. Local reductions in tuna stocks force seabirds to forage farther from shore, expending additional energy and making them more vulnerable to unregulated high seas fisheries. Masked boobies at Clipperton are now observed to forage more than three times farther offshore (430 nautical miles) (Jost et al., 2015), compared with ten years prior (Weimerskirch et al., 2009). The impacts of illegal visitation by sport fishers and divers is largely unknown. The endemic Clipperton angelfish (*Holacanthus limbaughi*) is highly prized in the aquarium fish trade and can be illegally sold for several thousand dollars in US markets and up to $10,000 in Asian markets (Clua et al., 2019), making this illegal fishery very lucrative.

Clipperton is important in the pathways of numerous highly migratory species (e.g., tuna, sharks, seabirds, billfish, marine mammals) (Jost, 2005; Jost et al., 2015). Despite its isolation, small size, and location relative to the great East Pacific Barrier, there is connectivity reported across these oceanographic barriers, which makes Clipperton of high scientific interest as a likely important stepping-stone of connectivity between the east and west tropical Pacific for some species (Lessios & Robertson, 2006). The presence of widely distributed Indo-Pacific zooxanthellate corals at Clipperton indicates that these NE Pacific Islands probably serve as a stepping stone for dispersal into the far eastern Pacific region (Glynn, Veron & Wellington, 1996).

The deeper depths at Clipperton Atoll may serve as a refuge from increasing water temperatures associated with climate change. Glynn, Veron & Wellington (1996) noted that that the coral reef community at Clipperton extends down to ca. 70 m and these deeper depths may allow thermally sensitive coral species to endure increasing shallow sea temperatures and help to recolonize shallow areas once environmental conditions become more favorable (Bridge et al., 2013; Smith et al., 2014). Since top predators (e.g., sharks, groupers, snappers, jacks) utilize a range of depths and habitats, deep-water refugia only provide partial protection for these species (Rocha et al., 2018). In addition, signs of heavy fishing pressure, sedimentation, coral bleaching, and invasive species have been observed on MCEs in the Pacific and Caribbean (Rocha et al., 2018). While species composition can be distinct among depth zones, the overall connectivity of the ecosystem from shallow to deep necessitates a whole-ecosystem approach to management (Baldwin, Tornabene & Robertson, 2018).

The recent creation of a no-take reserve 12 nautical miles around the atoll in November 2016 will help conserve the entire ecosystem from fishing, landing, and other external threats. There are currently 57 Mexican fishing boats (47 purse seiners and 10 longliners) authorized to fish within the French EEZ surrounding Clipperton, but outside of the recently created 12 nautical mile reserve.

## Conclusions

The results of this study highlight the unique marine ecosystem of Clipperton Atoll, particularly the deep-sea environments, which have been poorly studied prior to our expedition. The mixture of technologies, spanning a broad depth range, provided for an integrated examination of the biodiversity of this isolated atoll in the TEP. The relatively low diversity, high endemism and unique biogeography of Clipperton is of high scientific interest as it is likely an important stepping-stone of connectivity between the east and west tropical Pacific for some species. Its remoteness has also resulted in substantial illegal, unreported and under-reported fishing, which appears to have negative impacts on the overall health of the ecosystem. To help restore populations of sharks, tunas, and other pelagic species, there is an urgent need for better management of the entire EEZ, which could include a better vessel monitoring system, satellite-based vessel monitoring and surveillance, expansion of the existing 12 nautical mile protected area, and a greater research presence.

##  Supplemental Information

10.7717/peerj.7279/supp-1 Table S1 Invertebrate taxa recorded at Clipperton Atoll during 2016 surveys

10.7717/peerj.7279/supp-2 Table S2 Fish taxa recorded at Clipperton during 2016 surveys

10.7717/peerj.7279/supp-3 Figure S1 Plot of binomial model for invertebrate order occurrence by depth Blue bands are 95% confidence intervals around the slope estimate.

10.7717/peerj.7279/supp-4 Figure S2 New records of fishes at Clipperton Atoll during 2016 surveys

10.7717/peerj.7279/supp-5 Figure S3 Depth distribution of fish families at Clipperton during 2016 surveys

10.7717/peerj.7279/supp-6 Supplemental Information 1 Raw taxa density by depth, location, and survey method

## Additional Information and Declarations

### Field Study Permissions

The following information was supplied relating to field study approvals (i.e., approving body and any reference numbers): Permission to conduct this work was provided by the Haut-Commissariat de la Republique en Polynesie Francaise (HC167CABBSIRIMG).

### Data Availability

The following information was supplied regarding data availability: The raw data is available as a Supplemental File. The raw data provides density estimates (number per meter squared) for all taxa observed using underwater surveys, submersibles, and deep drop cameras. Data include date, latitude and longitude.
