# Supplementary material for: Marine biodiversity from zero to a thousand meters at Clipperton Atoll (Île de La Passion), Tropical Eastern Pacific
Source: PeerJ. 2019 Jul 16;7:e7279. doi: 10.7717/peerj.7279 (PMC6640628; doi:10.7717/peerj.7279)
Supplement: Table S1 [file peerj-07-7279-s001.docx]

Table S1. Invertebrate taxa recorded during surveys at Clipperton.

| Phylum | Class | Order | Family | Genus species |
| --- | --- | --- | --- | --- |
| Annelida | Polychaeta | Polychaeta |  |  |
| Arthropoda | Arthropoda | Arthropoda |  |  |
| Arthropoda | Malacostraca | Decapoda | Aristeidae |  |
| Arthropoda | Malacostraca | Decapoda | Calappidae |  |
| Arthropoda | Malacostraca | Decapoda | Calappidae | *Platymera gaudichaudii* |
| Arthropoda | Malacostraca | Decapoda | Epialtidae | *Stenocionops* sp. |
| Arthropoda | Malacostraca | Decapoda | Galatheidae | *Munida hispida* |
| Arthropoda | Malacostraca | Decapoda | Galatheidae |  |
| Arthropoda | Malacostraca | Decapoda | Lithodidae | *Lithodes nintokuae* |
| Arthropoda | Malacostraca | Decapoda |  |  |
| Arthropoda | Malacostraca | Decapoda |  |  |
| Arthropoda | Malacostraca | Mysidacea |  |  |
| Bryozoa | Bryozoa | Bryozoa |  |  |
| Cnidaria | Anthozoa | Actiniaria | Boloceroididae |  |
| Cnidaria | Anthozoa | Actiniaria | Liponematidae | *Liponema brevicornis* |
| Cnidaria | Anthozoa | Actiniaria |  |  |
| Cnidaria | Anthozoa | Alcyonacea | Acanthogorgiidae | *Acanthogorgia* sp. |
| Cnidaria | Anthozoa | Alcyonacea | Acanthogorgiidae |  |
| Cnidaria | Anthozoa | Alcyonacea | Coralliidae | *Corallium laauense* |
| Cnidaria | Anthozoa | Alcyonacea | Coralliidae | *Corallium* sp. |
| Cnidaria | Anthozoa | Alcyonacea | Gorgoniidae | *Leptogorgia styx* |
| Cnidaria | Anthozoa | Alcyonacea |  |  |
| Cnidaria | Anthozoa | Anthozoa |  |  |
| Cnidaria | Anthozoa | Antipatharia | Antipathidae | *Antipathes galapagensis* |
| Cnidaria | Anthozoa | Antipatharia | Antipathidae | *Antipathes spp.* |
| Cnidaria | Anthozoa | Antipatharia | Antipathidae | *Stichopathes* sp. |
| Cnidaria | Anthozoa | Antipatharia | Aphanipathidae |  |
| Cnidaria | Anthozoa | Antipatharia |  |  |
| Cnidaria | Anthozoa | Octocorallia | Acanthogorgiidae | *Acanthogorgia* |
| Cnidaria | Anthozoa | Scleractinia | Agariciidae | *Leptoseris incrustans* |
| Cnidaria | Anthozoa | Scleractinia | Agariciidae | *Leptoseris scabra* |
| Cnidaria | Anthozoa | Scleractinia | Agariciidae | *Pavona clavus* |
| Cnidaria | Anthozoa | Scleractinia | Agariciidae | *Pavona explanulata* |
| Cnidaria | Anthozoa | Scleractinia | Agariciidae | *Pavona gigantea* |
| Cnidaria | Anthozoa | Scleractinia | Agariciidae | *Pavona maldivensis* |

S1 Table continued. Invertebrate taxa recorded during surveys at Clipperton.

| Cnidaria | Anthozoa | Scleractinia | Agariciidae | *Pavona minuta* |
| --- | --- | --- | --- | --- |
| Cnidaria | Anthozoa | Scleractinia | Agariciidae | *Pavona varians* |
| Cnidaria | Anthozoa | Scleractinia | Caryophylliidae | *Caryophyllia* sp. |
| Cnidaria | Anthozoa | Scleractinia | Caryophylliidae | *Coenosmilia inordinata* |
| Cnidaria | Anthozoa | Scleractinia | Caryophylliidae |  |
| Cnidaria | Anthozoa | Scleractinia | Dendrophylliidae | *Tubastraea coccinea* |
| Cnidaria | Anthozoa | Scleractinia |  |  |
| Cnidaria | Anthozoa | Scleractinia | Pocilloporidae | *Pocillopora capitata* |
| Cnidaria | Anthozoa | Scleractinia | Pocilloporidae | *Pocillopora effusus* |
| Cnidaria | Anthozoa | Scleractinia | Pocilloporidae | *Pocillopora elegans* |
| Cnidaria | Anthozoa | Scleractinia | Pocilloporidae | *Pocillopora eydouxi* |
| Cnidaria | Anthozoa | Scleractinia | Pocilloporidae | *Pocillopora meandrina* |
| Cnidaria | Anthozoa | Scleractinia | Pocilloporidae | *Pocillopora verrucosa* |
| Cnidaria | Anthozoa | Scleractinia | Pocilloporidae | *Pocillopora woodjonesi* |
| Cnidaria | Anthozoa | Scleractinia | Poritidae | *Porites arnaudi* |
| Cnidaria | Anthozoa | Scleractinia | Poritidae | *Porites australiensis* |
| Cnidaria | Anthozoa | Scleractinia | Poritidae | *Porites lobata* |
| Cnidaria | Anthozoa | Scleractinia | Poritidae | *Porites lutea* |
| Cnidaria | Anthozoa | Alcyonacea | Gorgoniidae | *Leptogorgia styx* |
| Cnidaria | Cnidaria | Cnidaria |  |  |
| Cnidaria | Crinoidea | Comatulida | Charitometridae | *Glyptometra lateralis* |
| Cnidaria | Hydrozoa | Hydrozoa |  |  |
| Cnidaria | Hydrozoa | Siphonophorae |  |  |
| Cnidaria | Hydrozoa | Siphonophorae |  |  |
| Cnidaria | Scyphozoa | Scyphozoa |  |  |
| Cnidaria | Scyphozoa | Semaeostomeae | Ulmaridae | *Aurelia aurita* |
| Ctenophora | Ctenophora | Ctenophora |  |  |
| Echinodermata | Asteroidea | Paxillosida | Astropectinidae |  |
| Echinodermata | Asteroidea | Paxillosida | Astropectinidae |  |
| Echinodermata | Asteroidea | Spinulosida | Echinasteridae |  |
| Echinodermata | Echinoidea | Diadematoida | Diadematidae | *Echinothrix diadema* |
| Echinodermata | Echinoidea | Echinoidea |  |  |
| Echinodermata | Ophiuroidea | Ophiurida | Amphiuridae | *Ophiothrix galapagensis* |

S1 Table continued. Invertebrate taxa recorded during surveys at Clipperton.

| Mollusca | Cephalopoda | Octopoda |  |  |
| --- | --- | --- | --- | --- |
| Mollusca | Gastropoda | Gastropoda |  |  |

| Porifera | Demospongiae | Agelasida | Agelasidae |  |
| --- | --- | --- | --- | --- |
| Porifera | Hexactinellida | Hexactinellida |  |  |
| Porifera | Hexactinellida | Hexactinosida | Farreidae | *Farrea* *sp.* |
| Porifera | Porifera | Porifera |  |  |
