# Supplementary material for: Marine biodiversity from zero to a thousand meters at Clipperton Atoll (Île de La Passion), Tropical Eastern Pacific
Source: PeerJ. 2019 Jul 16;7:e7279. doi: 10.7717/peerj.7279 (PMC6640628; doi:10.7717/peerj.7279)
Supplement: Table S2 [file peerj-07-7279-s002.docx]

Table S2. Fish taxa recorded during surveys at Clipperton in 2016. Endemic species = *, new records = ^+^, qualitative surveys without depth records = ^#^.

| Taxa |
| --- |
| Carcharhiniformes |
| Scyliorhinidae |
| *Apristurus* sp.^+^ |
| Carcharhinidae |
| *Carcharhinus albimarginatus* |
| *Carcharhinus falciformis* |
| *Carcharhinus galapagensis* |
| *Galeocerdo cuvier* |
| *Triaenodon obesus* |
| Sphyrnidae |
| *Sphyrna lewini* |
| Squaliformes |
| Etmopteridae  *Etmopterus benchleyi*^+^ |
| Echinorhinidae |
| *Echinorhinus cookei^+^* |
| Myliobatiformes |
| Myliobatidae |
| *Mobula* sp. |
| Chimaeriformes |
| Chimaeridae |
| *Hydrolagus melanophasma*^+^ |
| Anguilliformes |
| Muraenidae |
| *Echidna nebulosa* |
| *Gymnomuraena zebra* |
| *Gymnothorax dovii* |
| *Gymnothorax flavimarginatus* |
| *Gymnothorax* sp. |
| Gonorynchiformes |
| Chanidae |
| *Chanos chanos*^#^ |
| Myctophiformes |
| Myctophidae |
| Gadiformes |
| Macrouridae |
| *Coryphaenoides* sp.^+^ |

Table S2 continued.

| Taxa |
| --- |
| Moridae |
| *Antimora* sp^+^ |
| Ophidiiformes |
| Ophidiidae |
| *Lamprogrammus cf. niger*^+^ |
| Bythitidae^+^ |
| Beryciformes |
| Holocentridae |
| *Myripristis berndti* |
| *Myripristis gildi** |
| *Myripristis* sp. |
| Syngnathiformes |
| Aulostomidae |
| *Aulostomus chinensis* |
| Fistulariidae |
| *Fistularia commersonii* |
| Scorpaeniformes |
| Scorpaenidae |
| *Pontinus* sp. A^+^ |
| *Pontinus vaughani* |
| Liparidae^+^ |
| Perciformes |
| Serranidae |
| *Dermatolepis dermatolepis* |
| *Epinephelus cifuentesi^+^* |
| *Epinephelus clippertonensis* |
| *Epinephelus labriformis* |
| *Epinephelus* sp. |
| *Liopropoma fasciatum* |
| *Paranthias colonus^#^* |
| Priacanthidae |
| *Cookeolus japonicus^+^* |
| *Heteropriacanthus cruentatus* |
| Carangidae |
| *Caranx lugubris* |
| *Caranx melampygus* |

Table S2 continued.

| Taxa |
| --- |
| *Caranx sexfasciatus^#^* |
| *Elagatis bipinnulata* |
| *Seriola dorsalis* |
| *Seriola rivioliana* |
| *Trachinotus stilbe* |
| Lutjanidae |
| *Lutjanus* sp. |
| *Lutjanus viridis* |
| Kyphosidae |
| *Kyphosus analogus* |
| *Kyphosus elegans* |
| *Kyphosus ocyurus* |
| Chaetodontidae |
| *Forcipiger flavissimus* |
| *Johnrandallia nigrirostris* |
| *Prognathodes carlhubbsi^+^* |
| Mullidae |
| *Mulloidichthys dentatus* |
| Pomacanthidae |
| *Holacanthus clarionensis* |
| *Holacanthus limbaughi** |
| Cirrhitidae |
| *Cirrhitichthys oxycephalus* |
| *Cirrhitus rivulatus* |
| Pomacentridae |
| *Chromis alta* |
| *Chromis* sp. |
| *Stegastes baldwini** |
| Labridae |
| *Bodianus diplotaenia* |
| *Novaculichthys taeniourus* |
| *Stethojulis bandanensis* |
| *Thalassoma grammaticum* |
| *Thalassoma purpureum* |
| *Thalassoma robertsoni** |
| *Thalassoma virens* |
| Scaridae |
| *Scarus rubroviolaceus* |

Table S2 continued.

| Taxa |
| --- |
| Blenniidae |
| *Ophioblennius steindachneri^#^* |
| Zanclidae |
| *Zanclus cornutus* |
| Acanthuridae |
| *Acanthurus guttatus^#^* |
| *Acanthurus nigricans* |
| *Acanthurus triostegus* |
| *Ctenochaetus marginatus* |
| *Naso lituratus^#^* |
| Sphyraenidae |
| *Sphyraena qenie* |
| *Sphyraena* sp. |
| Scombridae |
| *Acanthocybium solandri^#^* |
| *Euthynnus lineatus^#^* |
| *Katsuwonus pelamis* |
| *Scomber japonicus^+^* |
| *Thunnus albacares* |
| Pleuronectiformes |
| Bothidae |
| *Bothus mancus^#^* |
| Tetraodontiformes |
| Balistidae |
| *Balistes polylepis* |
| *Canthidermis maculatus+* |
| *Melichthys niger^#^* |
| *Sufflamen verres^#^* |
| Monacanthidae |
| *Aluterus scriptus#* |
| *Cantherhines dumerilii* |
| Ostraciidae |
| *Ostracion meleagris* |
| Tetraodontidae |
| *Arothron meleagris* |
| *Canthigaster punctatissima* |
| Diodontidae |
| *Chilomycterus reticulatus#* |
| *Diodon holocanthus* |
