# Supplementary figures and images for: Marine biodiversity from zero to a thousand meters at Clipperton Atoll (Île de La Passion), Tropical Eastern Pacific
Source: PeerJ. 2019 Jul 16;7:e7279. doi: 10.7717/peerj.7279 (PMC6640628; doi:10.7717/peerj.7279)

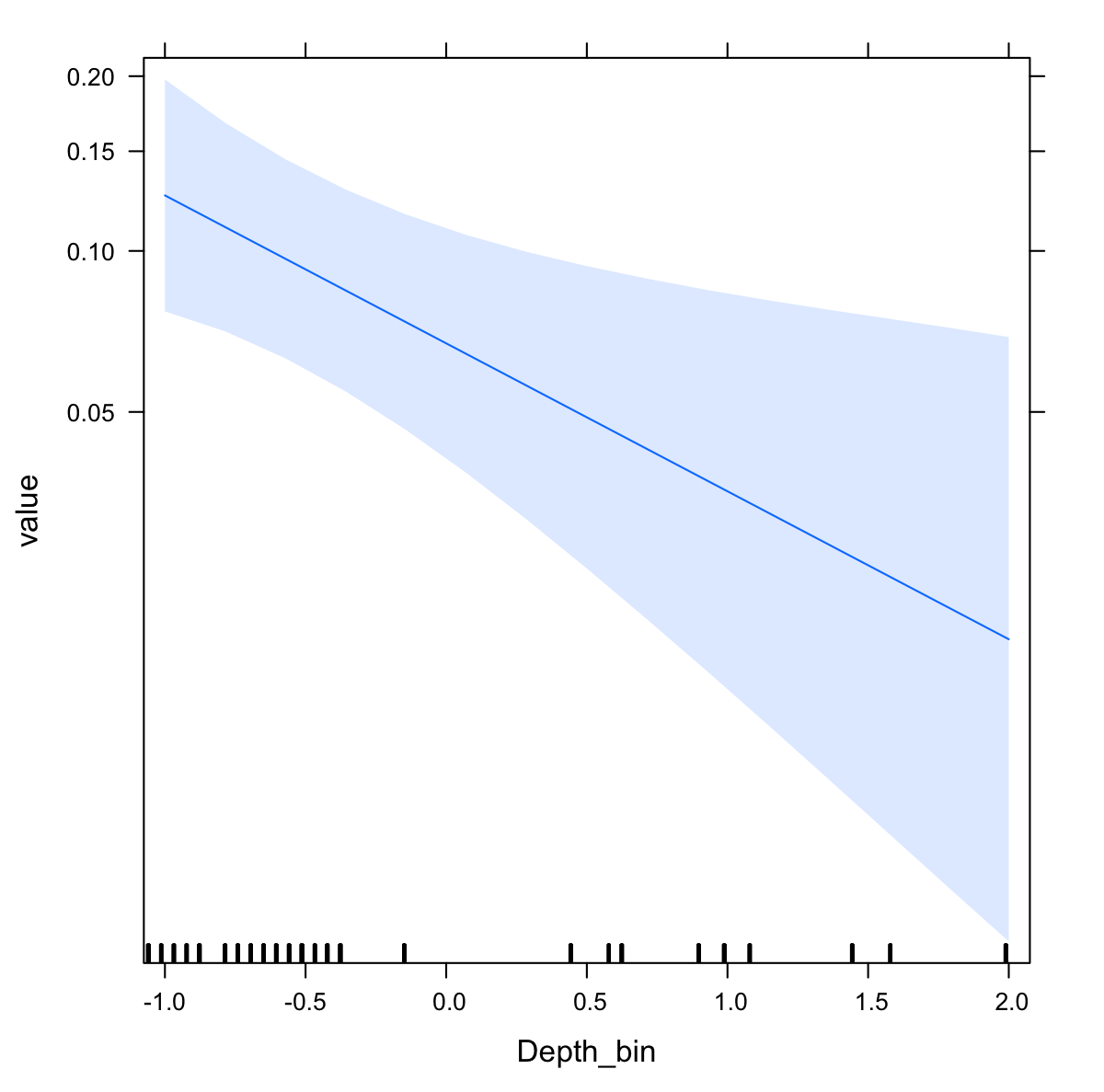

Supplement: Figure S1 — Blue bands are 95% confidence intervals around the slope estimate. [file peerj-07-7279-s003.png]

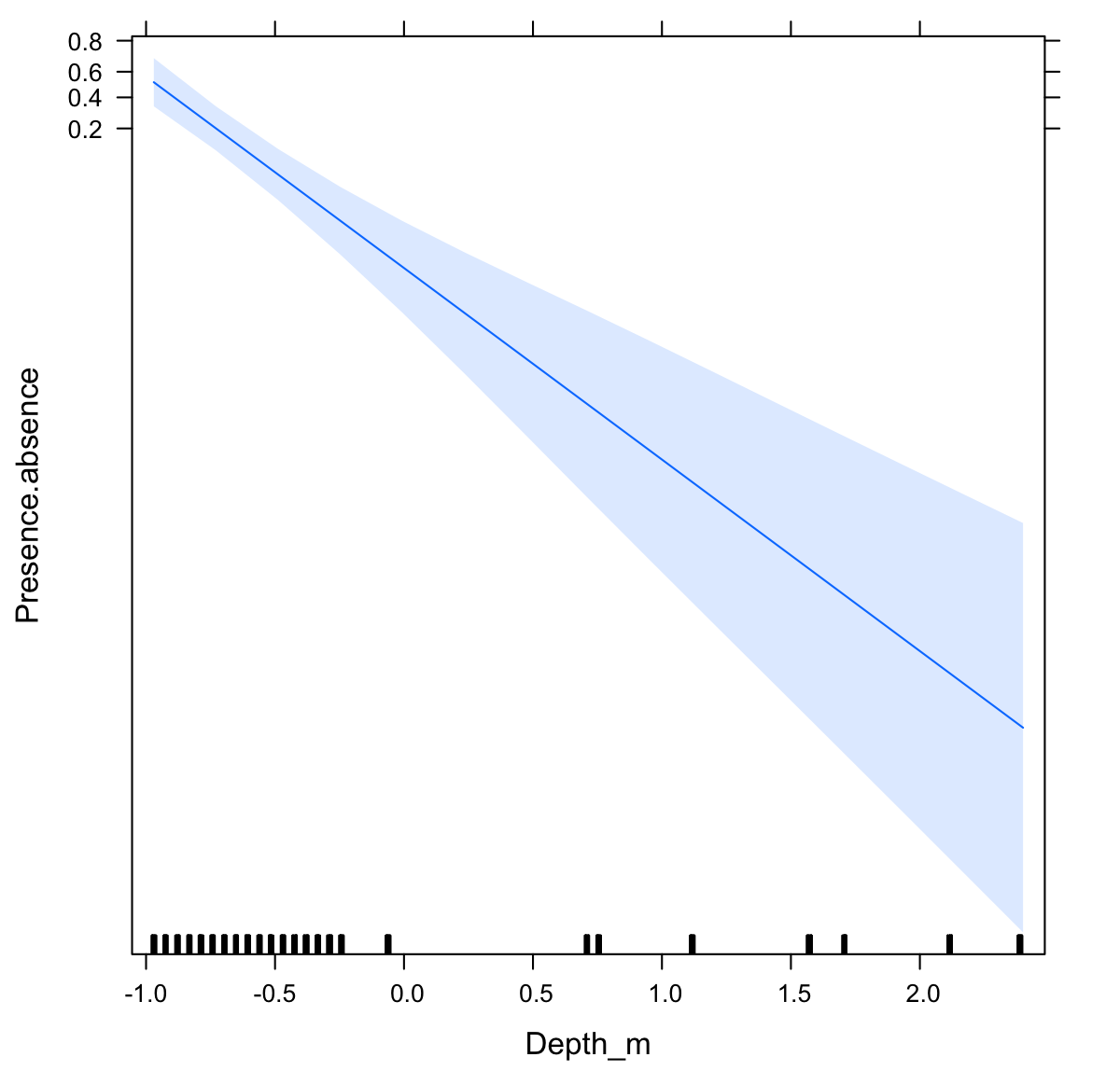

Supplement: Figure S3 [file peerj-07-7279-s005.png]
