# Supplementary material for: Marine biodiversity from zero to a thousand meters at Clipperton Atoll (Île de La Passion), Tropical Eastern Pacific
Source: PeerJ. 2019 Jul 16;7:e7279. doi: 10.7717/peerj.7279 (PMC6640628; doi:10.7717/peerj.7279)

Apristurus sp. – 1126 m.

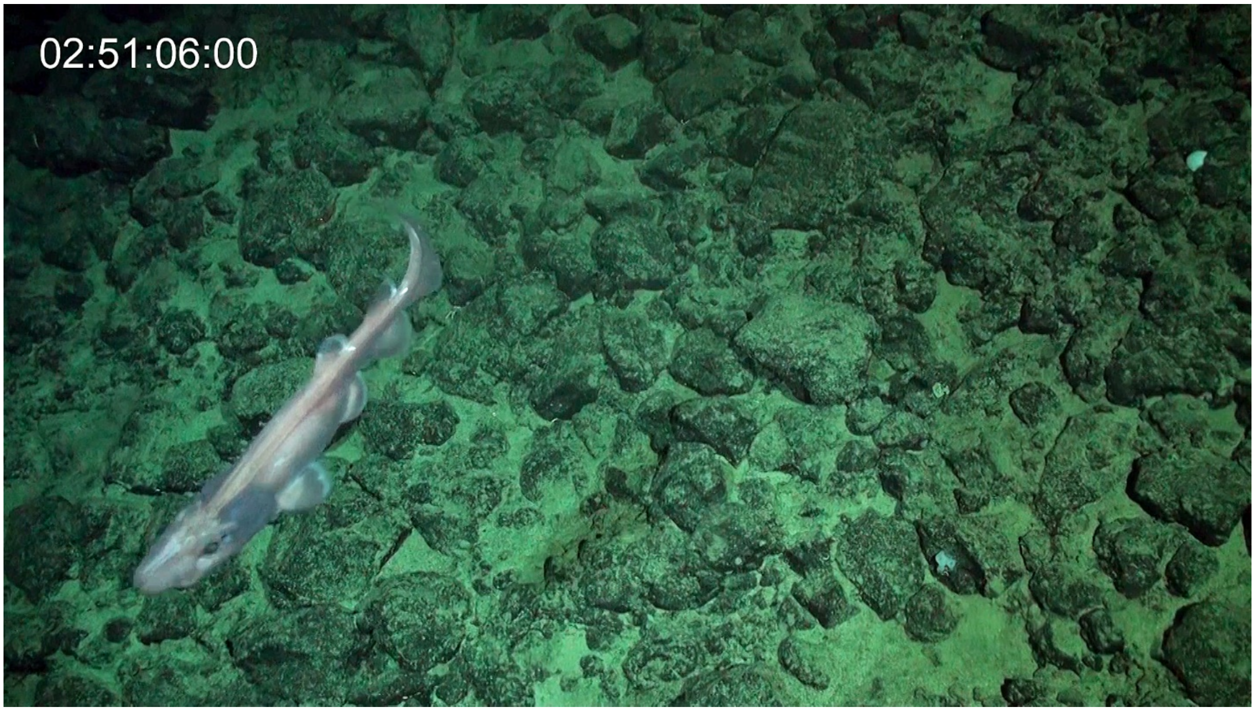

Hydrolagus melanophasma – 1126 m.

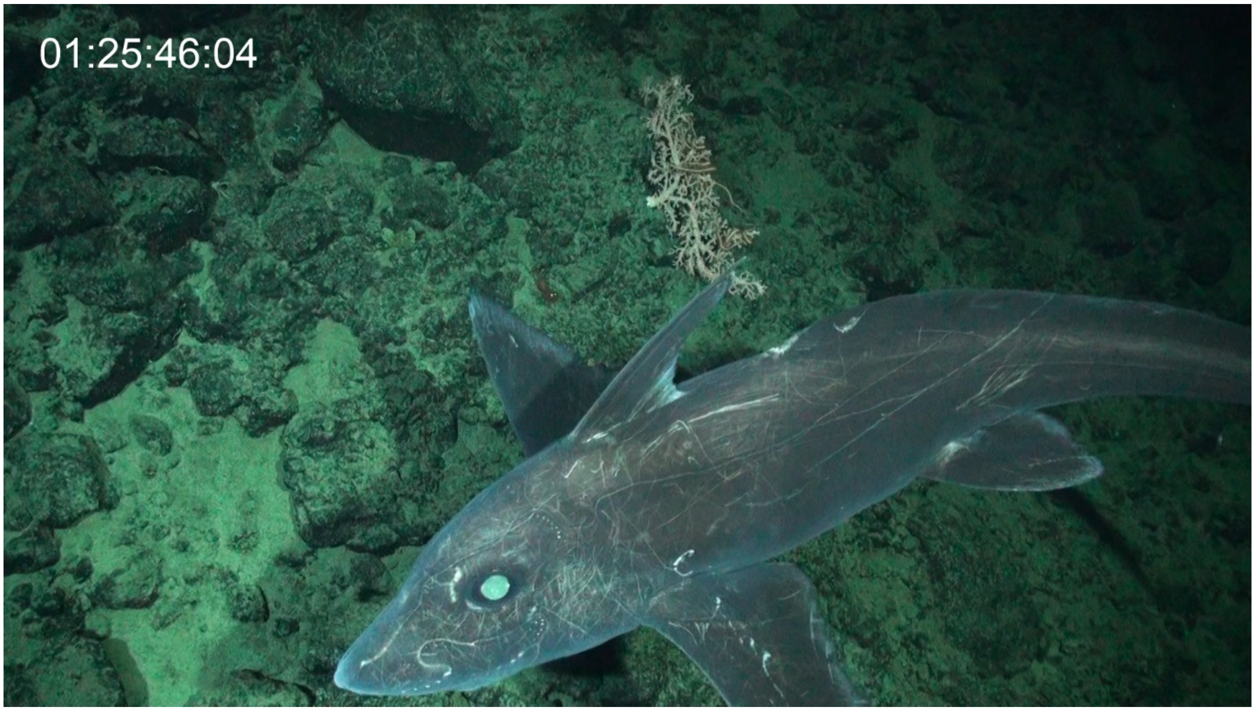

Myctophidae – 754 m.

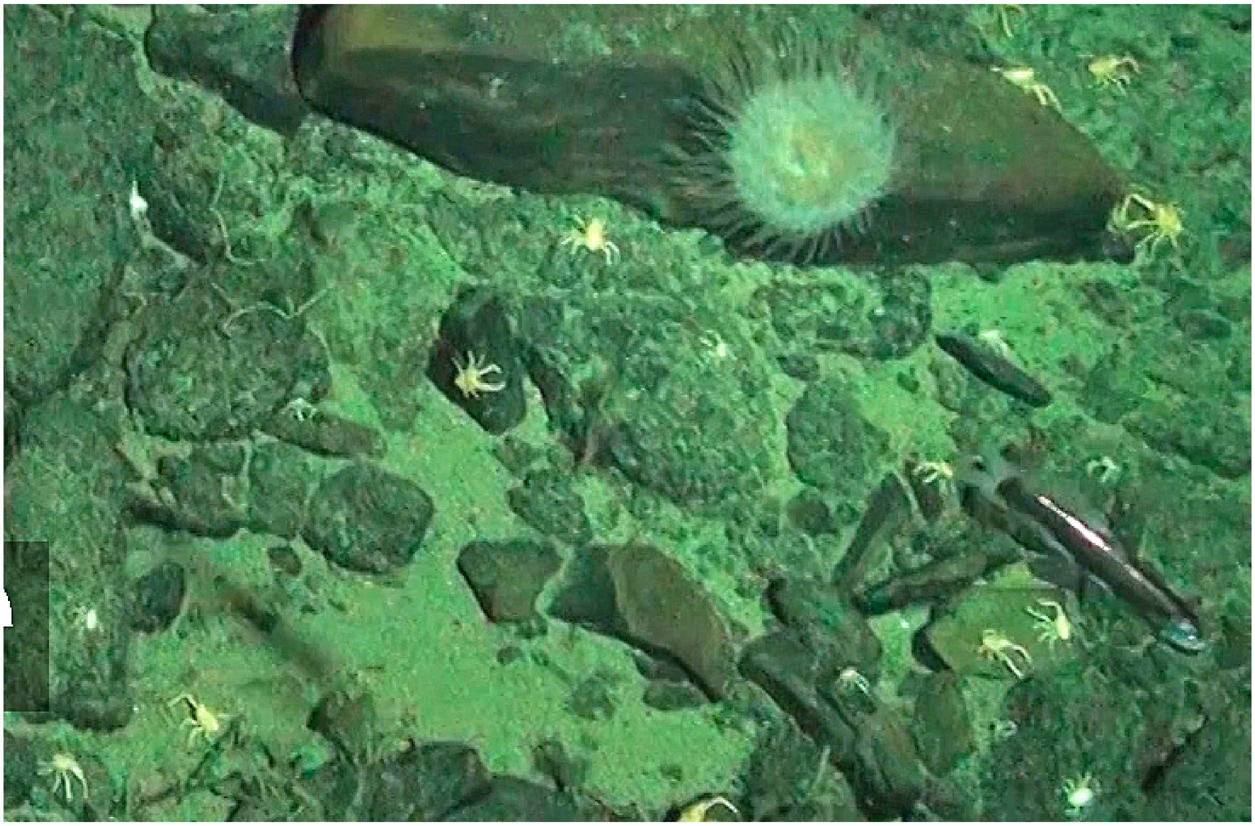

Coryphaenoides sp. – 1197 m.

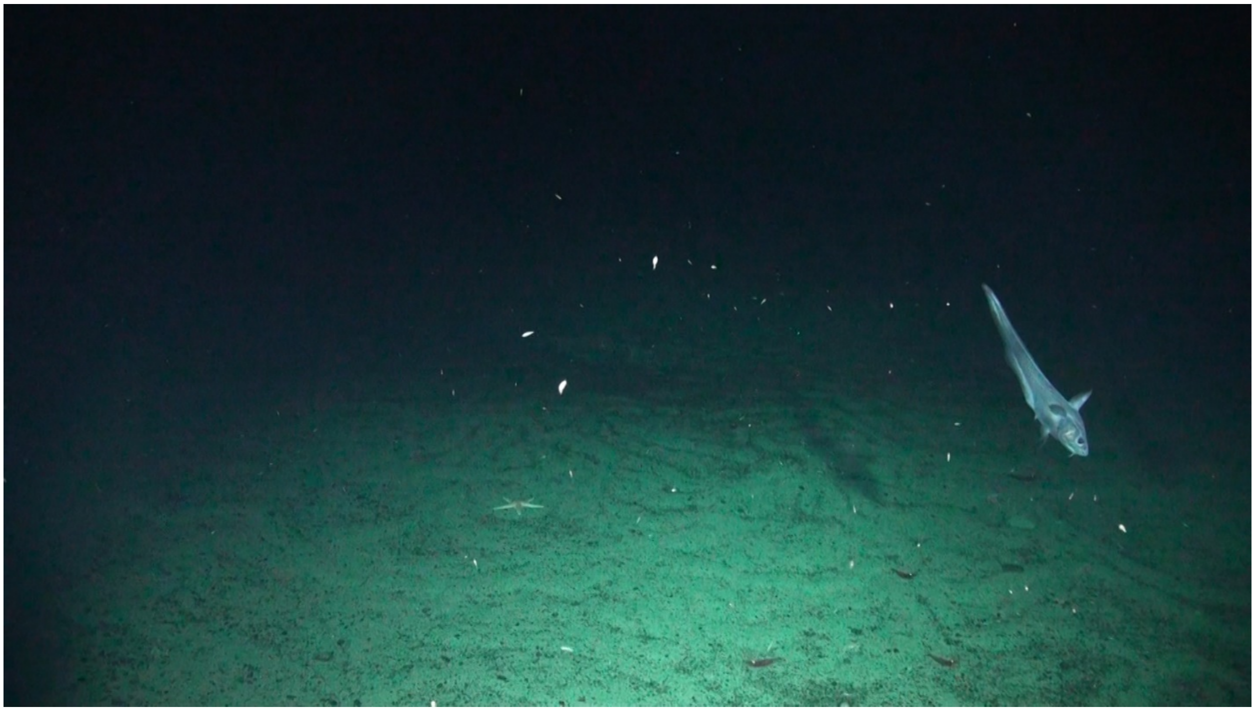

Lamprogrammus cf. *niger* – 967 m.

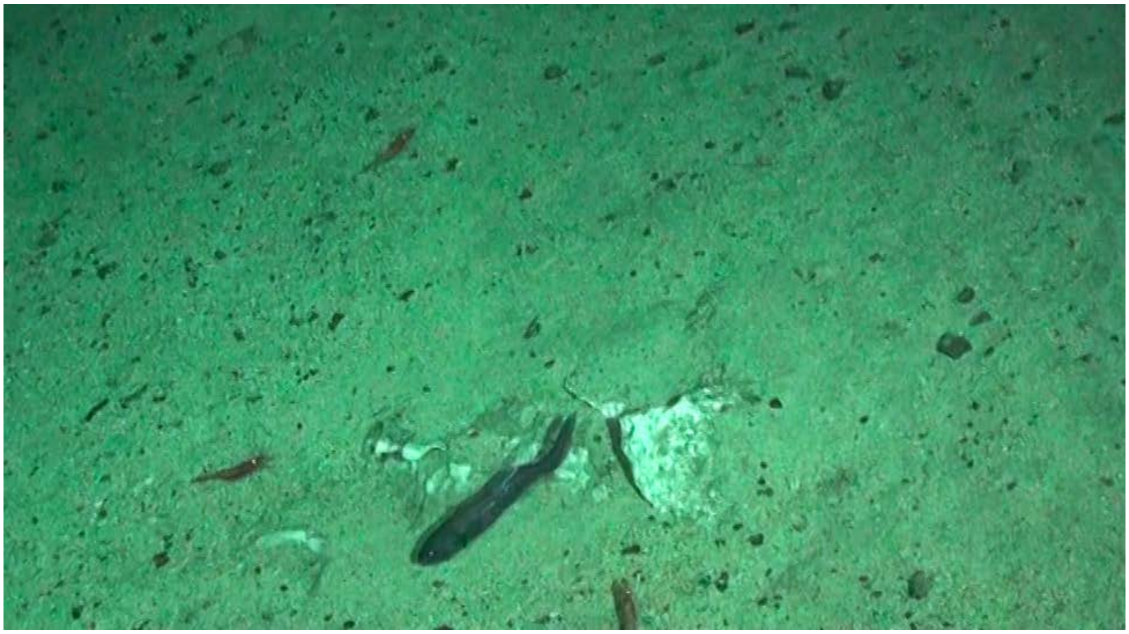

Pontinus sp. A. – 411 m.

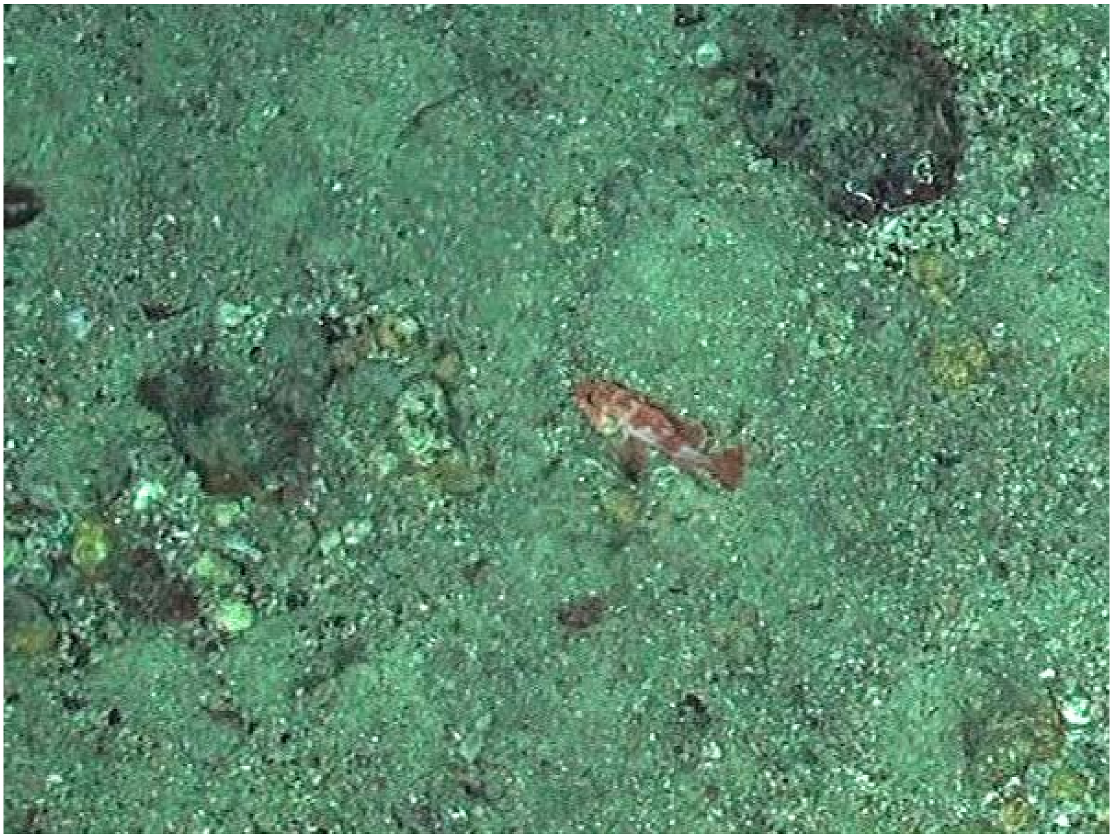

Pontinus sp. A – 270 m.

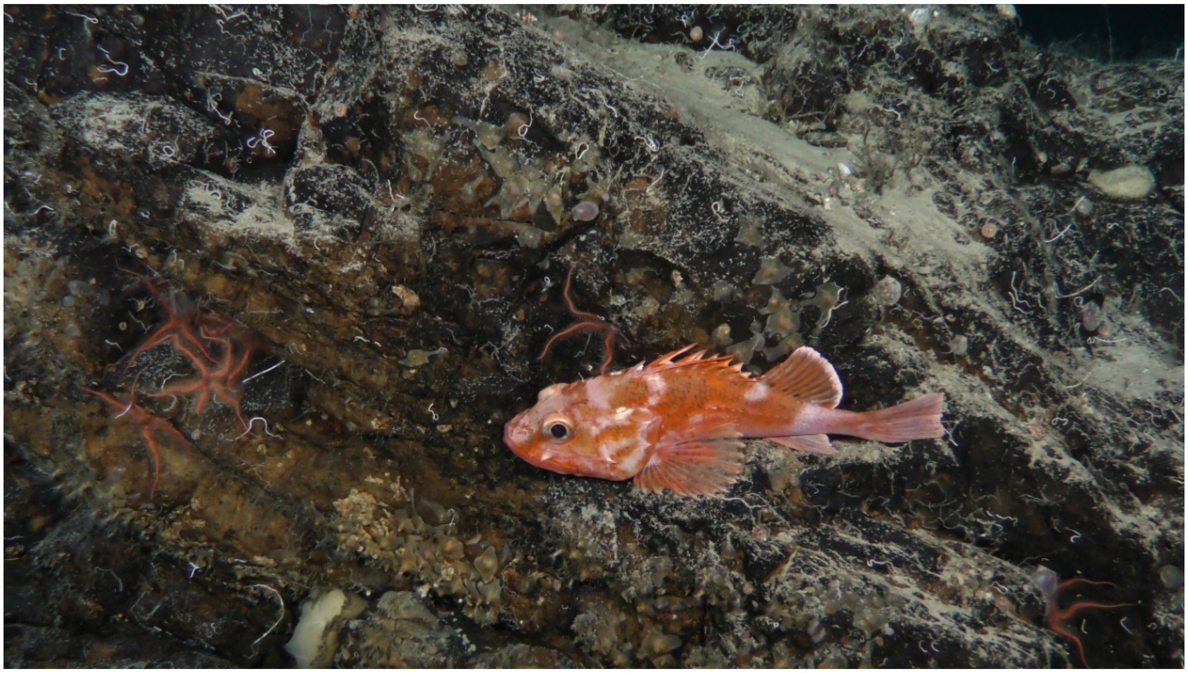

Liparidae – 1197 m.

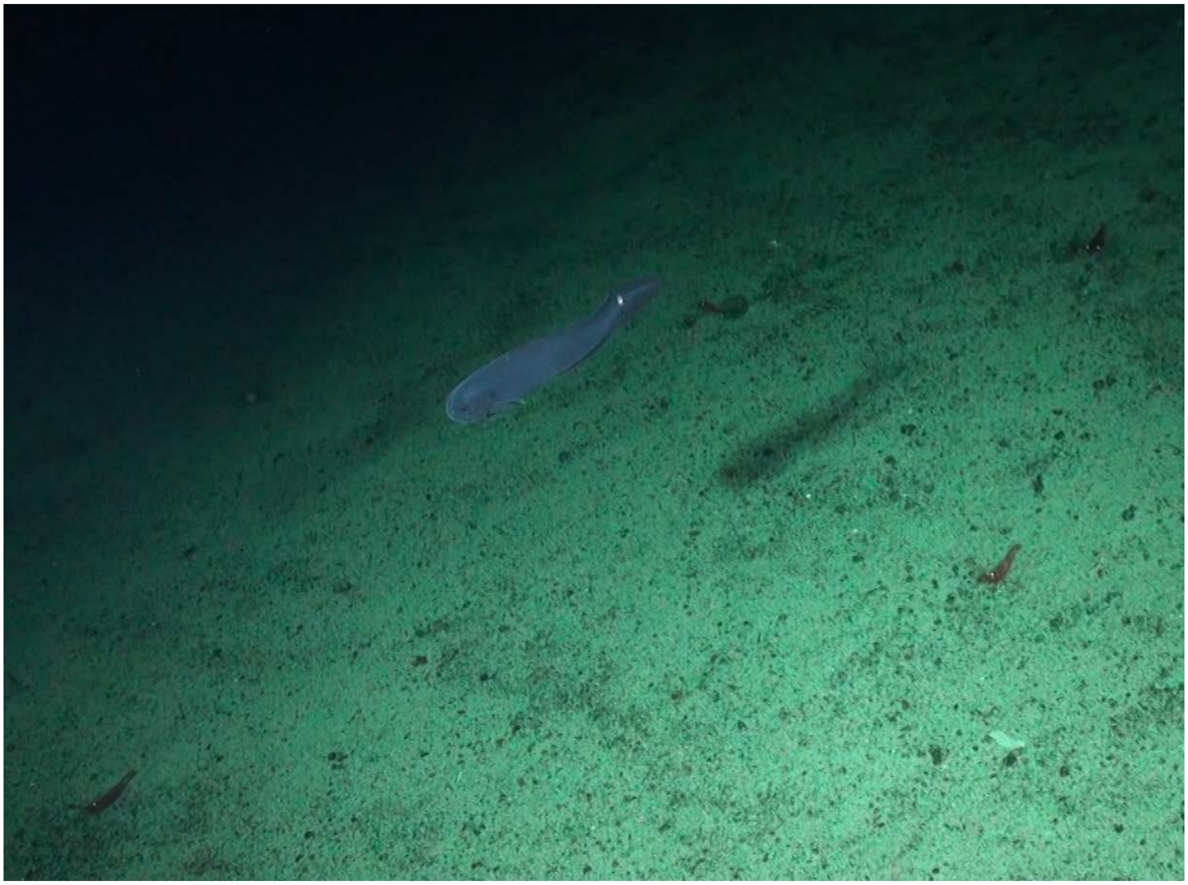

Epinephelus cifuentesi – 132 m.

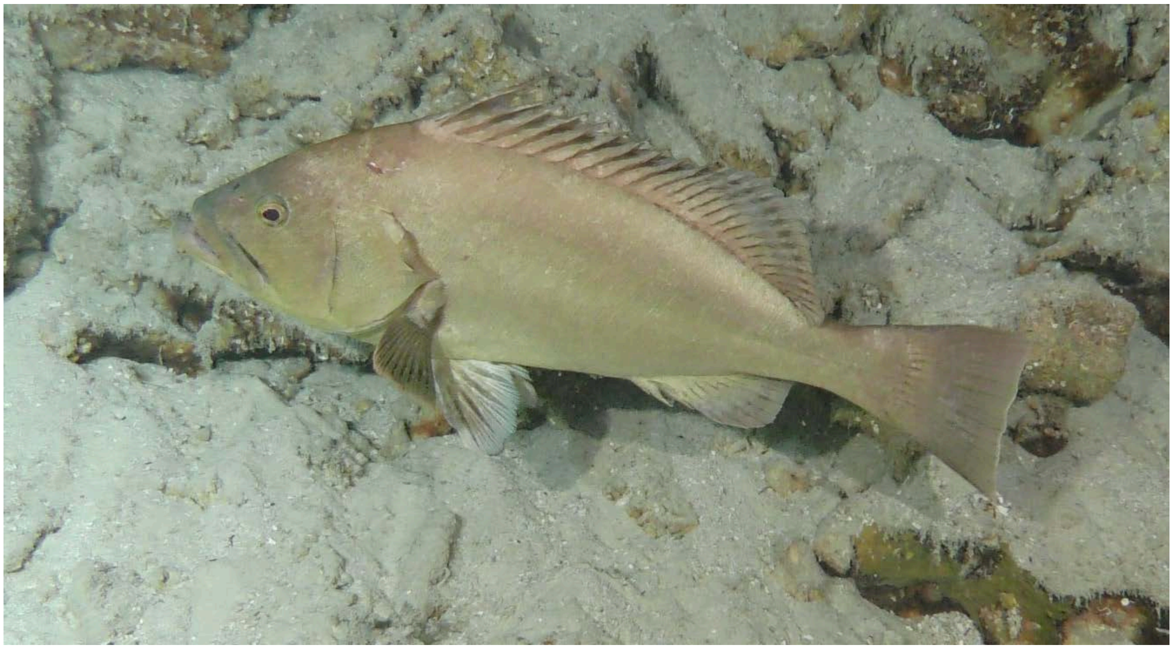

Prognathodes carlhubbsi – 126 m.

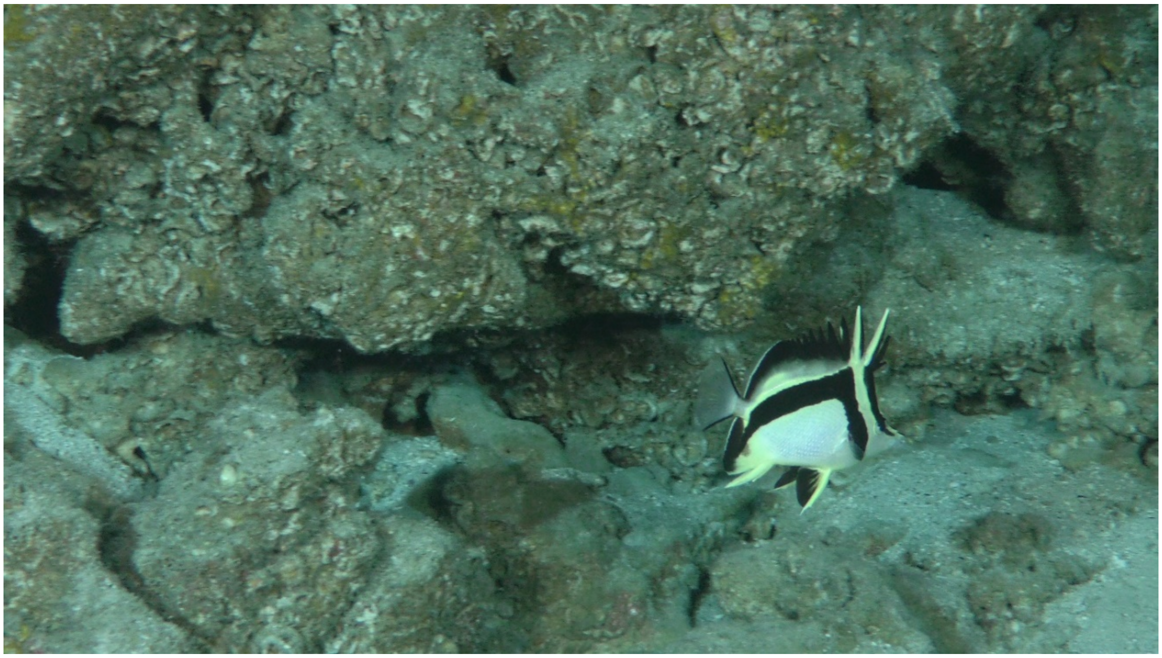

Supplement: Figure S2 [file peerj-07-7279-s004.pdf]
